# N6-methyladenosine facilitates mitochondrial fusion of colorectal cancer cells via induction of GSH synthesis and stabilization of OPA1 mRNA

**DOI:** 10.1093/nsr/nwae039

**Published:** 2024-01-29

**Authors:** Jiawang Zhou, Haisheng Zhang, Ke Zhong, Lijun Tao, Yu Lin, Guoyou Xie, Yonghuang Tan, You Wu, Yunqing Lu, Zhuojia Chen, Jiexin Li, Xin Deng, Qin Peng, Zigang Li, Hongsheng Wang

**Affiliations:** Guangdong Provincial Key Laboratory of New Drug Design and Evaluation, School of Pharmaceutical Sciences, Sun Yat-sen University, Guangzhou 510006, China; Guangdong Provincial Key Laboratory of New Drug Design and Evaluation, School of Pharmaceutical Sciences, Sun Yat-sen University, Guangzhou 510006, China; Guangdong Provincial Key Laboratory of New Drug Design and Evaluation, School of Pharmaceutical Sciences, Sun Yat-sen University, Guangzhou 510006, China; Guangdong Provincial Key Laboratory of New Drug Design and Evaluation, School of Pharmaceutical Sciences, Sun Yat-sen University, Guangzhou 510006, China; Guangdong Provincial Key Laboratory of New Drug Design and Evaluation, School of Pharmaceutical Sciences, Sun Yat-sen University, Guangzhou 510006, China; Guangdong Provincial Key Laboratory of New Drug Design and Evaluation, School of Pharmaceutical Sciences, Sun Yat-sen University, Guangzhou 510006, China; Guangdong Provincial Key Laboratory of New Drug Design and Evaluation, School of Pharmaceutical Sciences, Sun Yat-sen University, Guangzhou 510006, China; Guangdong Provincial Key Laboratory of New Drug Design and Evaluation, School of Pharmaceutical Sciences, Sun Yat-sen University, Guangzhou 510006, China; Guangdong Provincial Key Laboratory of New Drug Design and Evaluation, School of Pharmaceutical Sciences, Sun Yat-sen University, Guangzhou 510006, China; Sun Yat-sen University Cancer Center; State Key Laboratory of Oncology in South China and Collaborative Innovation Center for Cancer Medicine, Guangzhou 510060, China; Guangdong Provincial Key Laboratory of New Drug Design and Evaluation, School of Pharmaceutical Sciences, Sun Yat-sen University, Guangzhou 510006, China; Department of Biomedical Sciences, City University of Hong Kong, Hong Kong 999077, China; Institute of Systems and Physical Biology, Shenzhen Bay Laboratory, Shenzhen 518067, China; Institute of Systems and Physical Biology, Shenzhen Bay Laboratory, Shenzhen 518067, China; Guangdong Provincial Key Laboratory of New Drug Design and Evaluation, School of Pharmaceutical Sciences, Sun Yat-sen University, Guangzhou 510006, China

**Keywords:** m^6^A, mitochondrial fusion, glutathione, OPA1, colorectal cancer

## Abstract

Mitochondria undergo fission and fusion that are critical for cell survival and cancer development, while the regulatory factors for mitochondrial dynamics remain elusive. Herein we found that RNA m^6^A accelerated mitochondria fusion of colorectal cancer (CRC) cells. Metabolomics analysis and function studies indicated that m^6^A triggered the generation of glutathione (GSH) via the upregulation of RRM2B—a p53-inducible ribonucleotide reductase subunit with anti-reactive oxygen species potential. This in turn resulted in the mitochondria fusion of CRC cells. Mechanistically, m^6^A methylation of A1240 at 3′UTR of *RRM2B* increased its mRNA stability via binding with IGF2BP2. Similarly, m^6^A methylation of A2212 at the coding sequence (CDS) of OPA1—an essential GTPase protein for mitochondrial inner membrane fusion—also increased mRNA stability and triggered mitochondria fusion. Targeting m^6^A through the methyltransferase inhibitor STM2457 or the dm^6^ACRISPR system significantly suppressed mitochondria fusion. *In vivo* and clinical data confirmed the positive roles of the m^6^A/mitochondrial dynamics in tumor growth and CRC progression. Collectively, m^6^A promoted mitochondria fusion via induction of GSH synthesis and OPA1 expression, which facilitated cancer cell growth and CRC development.

## INTRODUCTION

Mitochondrial dynamics, which comprises mitochondrial fission and fusion, is a variation of mitochondrial morphology in response to different physiological conditions [1]. The fusion/fission process is regulated by homo- and heterotypic interactions between mitofusin 1 (MFN1) and MFN2 at the outer mitochondrial membrane and optic atrophy 1 (OPA1) at the inner mitochondrial membrane [2]. The function of mitochondrial fusion is critical for maintaining a healthy mitochondrial population [3]. For example, OPA1 is crucial for inner mitochondrial membrane (IMM) fusion and the arrangement of electron transport chain (ETC) super complexes, whilst loss of OPA1 causes the collapse of the mitochondrial network, impairs mitochondrial morphology and promotes apoptosis [4]. In addition, mitochondrial dynamics and bioenergetics reciprocally influence each other [5]. Increased oxidative phosphorylation (OXPHOS) activity can stimulate mitochondrial fusion to cause elongation [6].

Studies have revealed that dysfunction of mitochondrial dynamics is critical for cancer progression by disturbing cellular apoptosis, energy production and signal transduction [7,8]. With regard to colorectal cancer (CRC)—the third most prevalent cancer and the second most lethal malignancy in the world [9]—mitochondrial dynamics contributes to the pathogenesis of intestinal inflammation, colorectal tumorigenesis and CRC transformation [10]. For example, fatty acid-induced mitochondrial fission potentiated Wnt signaling in colon cancer to regulate *in vivo* formation of tumor organoids and growth of a xenograft tumor [11]. Hypoxia-induced activation of the OMA1–OPA1 axis increased mitochondrial reactive oxygen species (ROS) and promoted glycolysis in CRC cells [12]. Given that mitochondria dynamics plays a significant role in maintaining cellular structure and function, investigations into its regulatory factors will be greatly helpful for identification of therapy targets for cancer treatment.

*N* ^6^-methyladenosine (m^6^A) is the most abundant mRNA modification in eukaryotes that has been identified since the 1970s [13,14]. Dynamic m^6^A modification in a specific transcript is governed by m^6^A methyltransferase complexes composited by methyltransferase-like 3 (METTL3), METTL14, Wilms' tumor 1-associating protein (WTAP) and m^6^A demethylases fat mass and obesity-associated protein (FTO), and AlkB homolog 5 (ALKBH5) [15]. m^6^A modification of mRNA was recognized using m^6^A readers including YTH-domain family proteins (YTHDF1/2/3) and the insulin-like growth factor 2 (IGF2) mRNA-binding protein family (IGF2BP1/2/3) to regulate mRNA translation, cellular location and degradation, and modulate protein production [14,16]. As a fundamental regulatory factor for gene expression, m^6^A plays a role in various biological processes, including cell differentiation, embryonic development and cancer development [14].

Recent studies have identified a connection between mitochondrial dynamics and a number of epigenetic regulatory factors [17]. It has been reported that SIRT3-mediated deacetylation of OPA1 at K931, as well as a novel site at K834, is important in mediating mitochondrial elongation [18] while DNMT1 maintains the metabolic fitness of adipocytes through acting as an epigenetic safeguard of mitochondrial dynamics [19]. Little evidence has indicated that m^6^A may be critical for mitochondrial fission/fusion and metabolism. For example, FTO promotes growth and metastasis of gastric cancer via metabolic regulation of mitochondrial dynamics [20]. However, whether m^6^A modification could directly regulate mitochondrial fission/fusion and affect the metabolism of cancer cells remains unknown. In the present study, we investigated the potential effects of m^6^A on mitochondrial dynamics of CRC cells. Our data revealed that m^6^A can positively promote mitochondria fusion of CRC cells via induction of glutathione (GSH) synthesis and stabilization of *OPA1* mRNA.

## RESULTS

### m^6^A facilitated mitochondria fusion of CRC cells

**Figure 1.**
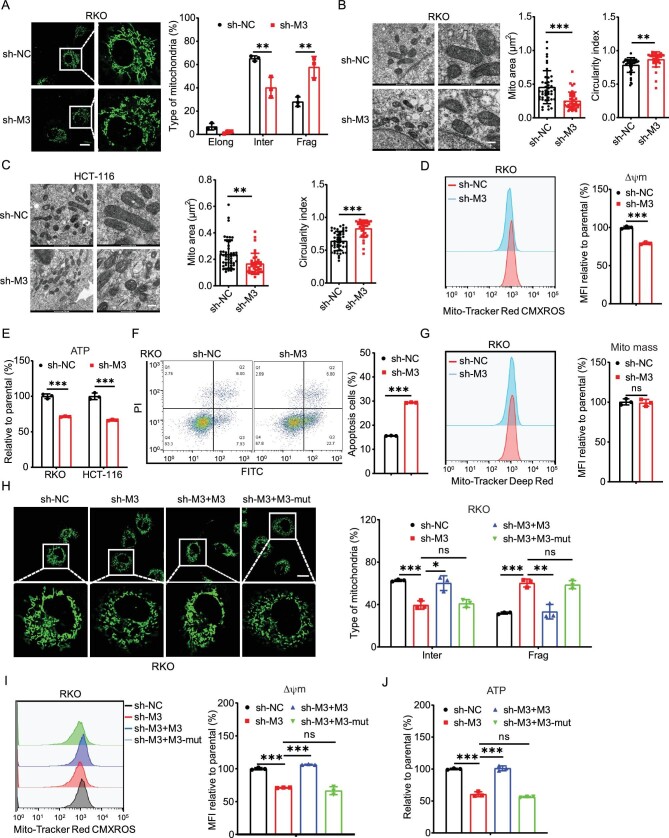
m^6^A facilitated mitochondria fusion of CRC cells. (A) Representative confocal images of mitochondrial morphology in sh-control (sh-NC) and sh-*METTL3* (sh-M3) RKO cells (left) and the percentages of mitochondrial types were measured (right). Elong, elongated type (˃3 μm); Inter, intermediate type (0.5–3 μm); Frag, fragmented type (˂0.5 μm) (scale bar, 20 μm). (B and C) Representative transmission electron microscopy images of the mitochondria in sh-control and sh-*METTL3* RKO (B) or HCT-116 (C) cells. The average mitochondrial area and circularity indexes were quantified via transmission electron microscope (TEM) analysis (scale bar, 200 nm). (D) Mitochondrial membrane potential (Δψm) was measured in sh-control and sh-*METTL3* RKO cells using flow cytometry (left) and median fluorescence intensity (MFI) was measured (right). (E) Intracellular ATP levels were determined via luciferin/luciferase-based assays in sh-control and sh-*METTL3* RKO and HCT-116 cells. (F) The percentages of apoptosis cells in sh-control and sh-*METTL3* RKO cells treated with 3 μM H_2_O_2_ for 4 h. (G) MitoTracker Deep Red staining was performed on sh-control and sh-*METTL3* RKO cells to stain the mitochondria for flow cytometric analysis (left) and MFI was measured (right). (H) Representative confocal images of the mitochondrial morphology in sh-control and sh-*METTL3* RKO cells transfected with vector control, METTL3 WT plasmid, METTL3 DA mutant plasmid for 24 h (scale bar, 20 μm). (I) Mitochondrial membrane potential in sh-control and sh-*METTL3* RKO cells transfected with vector control, METTL3 WT plasmid, METTL3 DA mutant plasmid for 24 h. (J) Intracellular ATP levels in sh-control and sh-*METTL3* RKO cells transfected with vector control, METTL3 WT plasmid, METTL3 DA mutant plasmid for 24 h. Data are presented as mean ± SD from three independent experiments. *P < 0.05; **P < 0.01; ***P < 0.001; ns, no significant, using Student's *t*-test between two groups and using one-way ANOVA followed by Bonferroni test for multiple comparisons.

To quantitatively assess the effects of m^6^A on mitochondrial homeostasis, we generated sh-control and sh-*METTL3* RKO (cell line name) and HCT-116 cells with lentiviruses (Fig. S1A). Staining with MitoTracker Green showed that sh-control RKO cells contained an extended network of elongated mitochondria uniformly distributed in the perinuclear region, while sh-*METTL3* cells had mostly smaller and shorter rounded mitochondria (Fig. 1A). Mitochondrial length was measured using ImageJ-MiNA and categorized as elongated (˃3 μm), intermediate (0.5–3 μm) or fragmented (˂0.5 μm) types [21]. Results showed that the fragmented mitochondria were increased significantly in sh-*METTL3* RKO cells (Fig. 1A). This phenomenon was similarly observed in HCT-116 cells (Fig. S1B). Furthermore, electron microscopy (EM) showed that sh-control RKO (Fig. 1B) and HCT-116 (Fig. 1C) cells displayed exaggerated mitochondrial elongation and swollen mitochondria in contrast to the sh-*METTL3* cells (*P* < 0.001). Statistical results indicated that sh-*METTL3* RKO or HCT-116 cells resulted in a 45% or 31% decrease in mitochondrial size in the corresponding control cells, which was associated with an increase in the circularity index (from 0.79 ± 0.02 to 0.87 ± 0.02, 0.64 ± 0.02 to 0.83 ± 0.02, respectively) (Fig. 1B and C). In addition, knock-down of METTL3 in RKO cells decreased mitochondrial cristae and caused a relative increase in abnormal mitochondrial cristae that were crooked and disordered (Fig. S1C). These data indicated that knock-down of METTL3 modulated the mitochondrial morphology in CRC cells.

Mitochondrial fusion is critical for mitochondrial function and is associated with increased oxidative phosphorylation [22]. Flow cytometer results indicated that sh-*METTL3* reduced the mitochondrial membrane potential (Δψm) in CRC cells (Fig. 1D and Fig. S1D). Moreover, knock-down of METTL3 resulted in a 29% and 34% decrease in adenosine triphosphate (ATP) levels in sh-*METTL3* RKO and HCT-116 cells, respectively (Fig. 1E). Further, the sh-*METTL3* RKO cells were significantly more sensitive to H_2_O_2_-induced cell apoptosis than the sh-control cells (Fig. 1F). This indicated that sh-*METTL3* induced significant changes in mitochondrial function.

We subsequently aimed to determine whether these changes were attributable to mitochondrial biogenesis by the use of MitoTracker Deep Red assay. The flow cytometry results showed that sh-*METTL3* elicited no significant changes in mitochondrial mass in the corresponding control cells (Fig. 1G and Fig. S1E). Consistently, mitochondrial DNA contents were comparable between sh-control and sh-*METTL3* CRC cells (Fig. S1F). This suggested that METTL3 had no effect on mitochondrial biogenesis.

To confirm the essential roles of m^6^A in mitochondrial dynamics, sh-*METTL3* RKO cells were transfected with wild-type (WT) METTL3 and catalytically inactive METTL3 mutant DA (D395A) [23] (Fig. S1G). Results showed that the overexpression of METTL3, rather than METTL3 DA mutant, reversed the mitochondrial size (Fig. 1H), membrane potential (Fig. 1I) and ATP levels (Fig. 1J) in RKO cells. Consistently, overexpression of the m^6^A demethylase ALKBH5 [24] (Fig. S1H) had similar effects on sh-*METTL3*, which resulted in a decrease in mitochondrial size (Fig. S1I), membrane potential (Fig. S1J) and ATP (Fig. S1K) in RKO cells. All these data suggested that m^6^A facilitated mitochondria fusion of CRC cells.

### Glutathione was involved in m^6^A-induced mitochondrial fusion

**Figure 2.**
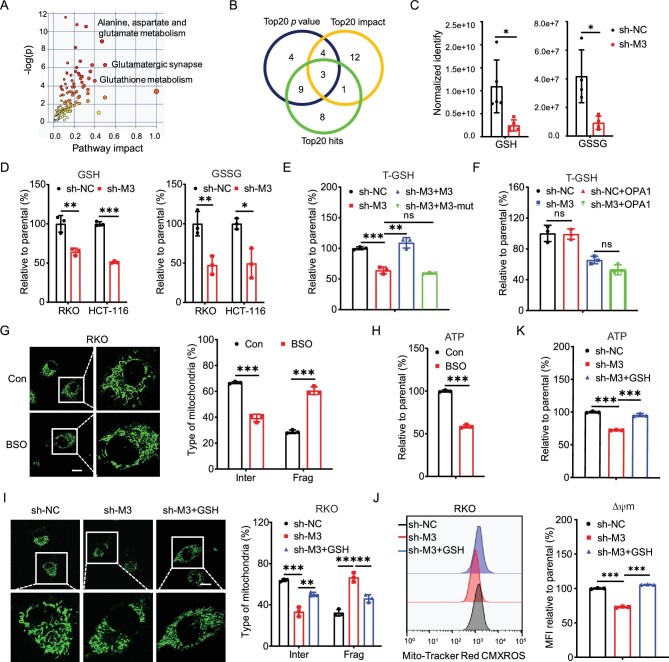
Glutathione was involved in m^6^A-induced mitochondrial fusion. (A) KEGG enrichment analysis of these 110 metabolites variated in sh-*METTL3* RKO cells. (B) The overlap among ‘Top 20 *P*-value’, ‘Top 20 impact’ and ‘Top 20 hits’ of the KEGG enrichment pathways. (C) The GSH and GSSG levels in sh-control and sh-*METTL3* RKO cells using the metabolomics analysis. (D) The GSH and GSSG levels in sh-control and sh-*METTL3* RKO or HCT-116 cells. (E) The total GSH (T-GSH, GSH + GSSG) levels in sh-control and sh-*METTL3* RKO cells transfected with vector control, METTL3 WT plasmid, METTL3 DA mutant plasmid for 24 h. (F) The T-GSH levels in sh-control or sh-*METTL3* RKO cells transfected with vector control or OPA1 plasmid for 24 h. (G and H) Representative confocal images of the mitochondrial morphology (G) and intracellular ATP levels (H) in RKO cells treated with 100 μM BSO for 24 h (scale bar, 20 μm). (I–K) Representative confocal images of the mitochondrial morphology (I), mitochondrial membrane potential (J) and intracellular ATP levels (K) in sh-*METTL3* RKO cells that were treated with 100 μM GSH for 24 h (scale bar, 20 μm). Data are presented as mean ± SD from three independent experiments. *P < 0.05; **P < 0.01; ***P < 0.001; ns, no significant, using Student's *t*-test between two groups and using one-way ANOVA followed by Bonferroni test for multiple comparisons.

Considering that mitochondrial dysfunction may alter cellular metabolites [25], we performed metabolomics analysis in sh-*METTL3* RKO cells by using gas chromatography–mass spectrometry (GC–MS) and carrying out multivariate statistical analysis using Principal Component Analysis (PCA), Partial Least Squares Discrimination Analysis (PLS-DA) and Orthogonal PLS-DA (OPLS-DA) to evaluate the metabolic pattern changes (Fig. S2A). By analysing using PCA, PLS-DA and OPLS-DA, we could markedly distinguish sh-*METTL3* cells from sh-control cells (Fig. S2A). In total, we identified 110 metabolites with significant changes in the *METTL3* knock-down RKO cells (Fig. S2B and Table S1). Kyoto Encyclopedia of Genes and Genomes (KEGG) enrichment analysis of these metabolites attributed 155 metabolic-related pathways in sh-*METTL3* RKO cells (Fig. 2A and Table S2). Among the identified pathways, glutathione metabolism, GABAergic synapse, and alanine, aspartate and glutamate metabolism were identified as the major target pathways in the METTL3-mediating metabolism of RKO cells via overlapping analysis of the top 20 *P*-values, impacts and hits (Fig. 2B). Constantly, metabolites in the glutathione metabolism pathway such as GSH, oxydized glutathione (GSSG), L-glutamic acid and ascorbate decreased, while spermidine and gamma-Glutamylalanine increased, which suggested reduced GSH generation in sh-*METTL3* RKO cells (Fig. 2C and Fig. S2C). GSH and GSSG Assay Kit analysis confirmed that the knock-down of METTL3 resulted in a significant decrease in GSH and GSSG levels in both RKO and HCT-116 cells (Fig. 2D).

Further, sh-*METTL3* RKO cells were transfected with WT METTL3 and catalytically inactive METTL3 mutant DA (D395A). Results showed that the overexpression of METTL3, rather than METTL3 DA mutant, reversed the total GSH (T-GSH, GSSG + GSH), GSH, GSSG levels in sh-*METTL3* RKO cells (Fig. 2E and Fig. S2D) while overexpression of ALKBH5 suppressed the levels of T-GSH in RKO cells (Fig. S2E). This indicated that m^6^A positively regulated the generation of GSH in CRC cells.

Mitochondria is affected by GSH redox changes, as mitochondria cannot produce GSH themselves and an accumulation of GSSG can cause widespread oxidation of proteins including essential mitochondrial proteins of the ETC [26,27]. At the same time, mitochondrial networks are readily responsive to redox imbalances [28], which in turn may reduce the production of ROS or protect mitochondria from degradation by mitophagy [29]. In order to evaluate the cause relationship between GSH generation and mitochondria fusion, both sh-control and sh-*METTL3* RKO cells were transfected with OPA1 (Fig. S2F). OPA1 regulates mitochondrial fusion and cristae structure in the IMM [30]. Overexpression of OPA1 affected GSH redox change (Fig. S2G), but had no significant effect on levels of T-GSH in either sh-control or sh-*METTL3* RKO cells (Fig. 2F), suggesting that mitochondrial dynamics had no effect on GSH generation.

Recent research demonstrated that GSSG strongly induces mitochondrial fusion by the generation of disulphide-mediated mitofusin oligomers, with Guanosine triphosphate (GTP) hydrolysis [31]. The addition of GSSG consistently reversed sh-*METTL3-*suppressed mitochondria fusion (Fig. S2H). To test whether reduced GSH generation leads to a decrease in GSSG production, ultimately inducing mitochondrial fission in CRC cells, we used L*-*buthionine-sulfoximine (BSO) to block GSH synthesis [32]. Our data showed that BSO suppressed mitochondria fusion (Fig. 2G) and ATP generation (Fig. 2H) in RKO cells. Further, the addition of GSH reversed sh-*METTL3-*suppressed mitochondria fusion (Fig. 2I), membrane potential (Fig. 2J) and ATP generation (Fig. 2K) in RKO cells. All these data indicated that m^6^A triggers GSH generation to induce mitochondria fusion.

### m^6^A-regulated GSH synthesis through stabilization of *RRM2B* mRNA

**Figure 3.**
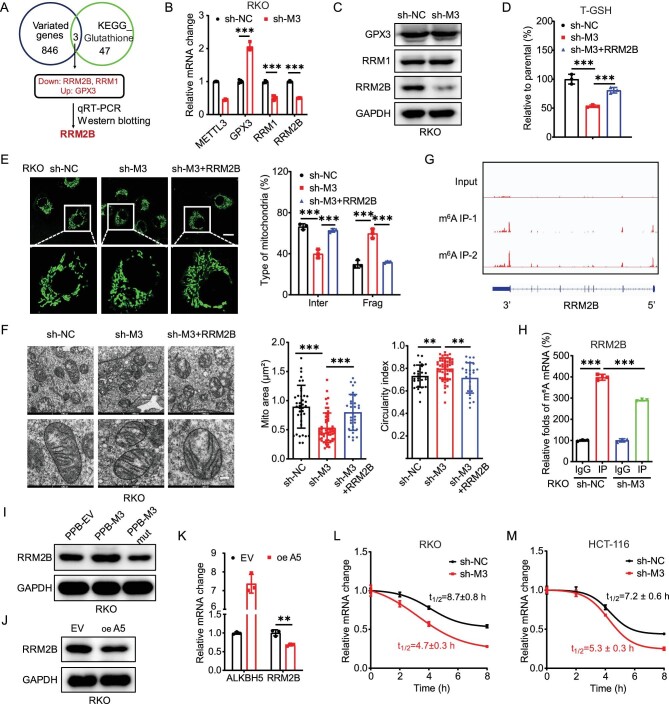
m^6^A-regulated GSH synthesis through stabilization of *RRM2B* mRNA. (A) The analysis process diagram to identify RRM2B as METTL3-regulated targets that were involved in glutathione metabolism pathways in CRC cells. (B and C) The mRNA (B) and protein (C) levels of RRM2B, RRM1 and GPX3 in sh-control and sh-*METTL3* RKO cells. (D and E) The T-GSH levels (D) and representative confocal images of the mitochondrial morphology (E) in sh-control and sh-*METTL3* RKO cells transfected with vector control or RRM2B plasmid for 24 h. (F) Representative transmission electron microscopy images of the mitochondria in sh-control, sh-*METTL3* and RRM2B stable overexpression sh-*METTL3* RKO cells. The average mitochondrial area and circularity indexes were quantified via TEM analysis (scale bar, 200 nm). (G) m^6^A peaks were enriched in *RRM2B* mRNA from m^6^A RIP-seq data. (H) m^6^A RIP–qPCR analysis of RRM2B in sh-control and sh-*METTL3* RKO cells. (I) The protein expression of RRM2B in RKO cells transfected with vector control, METTL3 WT plasmid or METTL3 DA mutant plasmid for 24 h. (J and K) The protein (J) and mRNA (K) expression of RRM2B in RKO cells transfected with vector control or ALKBH5 plasmid for 24 h. (L and M) After treatment with Act-D for the indicated times, the mRNA levels of *RRM2B* were checked in sh-control and sh-*METTL3* RKO (L) or HCT-116 (M) cells. Data are presented as mean ± SD from three independent experiments. *P < 0.05; **P < 0.01; ***P < 0.001; ns, no significant, using Student's *t*-test between two groups and using one-way ANOVA followed by Bonferroni test for multiple comparisons.

To identify potential targets involved in m^6^A-regulated GSH synthesis and fusion of mitochondria, we performed mRNA-seq in sh-control and sh-*METTL3* RKO cells. Expression levels of 849 genes were found to be significantly changed with the upregulation of 97 and downregulation of 752 genes in sh-*METTL3* RKO cells (Fig. S3A and Table S3). The genes varied in sh-*METTL3* RKO cells and those involved in glutathione metabolism (Table S4, summarized using Gene Set Enrichment Analysis (GSEA)) [33] were overlap analysed. Overlap analysis showed that three candidates (down: RRM2B (ribonucleotide reductase regulatory TP53 inducible subunit M2B) and RRM1 (ribonucleotide reductase catalytic subunit M1), up: GPX3 (glutathione peroxidase 3)) were overlapping between the gene set and variated genes in sh-*METTL3* RKO cells (Fig. 3A). qRT–PCR consistently showed that a reduction in METTL3 can increase the mRNA of *GPX3* and decrease the mRNA of *RRM2B* and *RRM1* in both RKO (Fig. 3B) and HCT-116 (Fig. S3B) cells. However, Western blot analysis showed that knock-down of METTL3 decreased the protein expression of RRM2B, while it had no consistent effect on GPX3 or RRM1, in RKO (Fig. 3C) and HCT-116 (Fig. S3C) cells.

RRM2B (also known as p53R2) is a p53-inducible ribonucleotide reductase subunit that exhibits anti-ROS potential [34]. We therefore investigated its roles in m^6^A-regulated GSH generation and mitochondria fusion of CRC cells. Results showed that overexpression of RRM2B (Fig. S3D) can reverse the downregulation of T-GSH levels (Fig. 3D) and mitochondria fusion (Fig. 3E and F) in sh-*METTL3* RKO cells. It indicated the essential roles of RRM2B in m^6^A-regulated GSH generation and mitochondria fusion of CRC cells.

We further investigated whether m^6^A regulated the expression of RRM2B via direct methylation of mRNA. m^6^A-RIP-seq data showed that the 3′UTR region of *RRM2B* mRNA was modified by m^6^A (Fig. 3G). m^6^A-RIP–qPCR confirmed that a 4-fold enrichment of m^6^A antibody was observed in *RRM2B* mRNA in RKO cells and this enrichment significantly decreased in sh-*METTL3* RKO cells (Fig. 3H). Similar results were also observed in HCT-116 cells (Fig. S3E). Furthermore, the overexpression of WT METTL3, but not the METTL3 DA mutant, reversed the levels of RRM2B in RKO cells (Fig. 3I) while the overexpression of ALKBH5 suppressed the protein expression of RRM2B in RKO cells (Fig. 3J). This indicated that RRM2B was modified by m^6^A and that m^6^A positively regulates the expression of RRM2B in CRC cells.

We further investigated the potential mechanisms involved in the m^6^A-regulated expression of RRM2B. To determine whether METTL3 can modulate the transcription of RRM2B, we performed a luciferase reporter assay by transfecting the promoter reporter gene plasmid pGL3-Basic-*RRM2B*-luc into RKO cells. There was no significant difference in the luciferase activity of the RRM2B promoter between sh-control and sh-METTL3 cells (Fig. S3F), suggesting that METTL3 does not affect the transcription of *RRM2B.* This was further confirmed using qRT–PCR analysis, which showed comparable levels of the precursor mRNA of RRM2B between sh-control and sh-METTL3 CRC cells (Fig. S3G). Additionally, fractionation assay results indicated that there was no difference in the subcellular localization of *RRM2B* mRNA in sh-control and sh-*METTL3* RKO cells (Fig. S3H). This indicated that m^6^A had no effect on the subcellular localization of *RRM2B* mRNA.

However, knock-down of METTL3 (Fig. 3B) or overexpression of ALKBH5 (Fig. 3K) significantly decreased the mRNA expression of RRM2B in CRC cells. Since m^6^A had no effect on promoter activity but regulated the mRNA expression of RRM2B, we then tested its effect on mRNA stability. Our results showed that knock-down of METTL3 significantly decreased the mRNA stability of RRM2B in both RKO (Fig. 3L) and HCT-116 (Fig. 3M) cells. Therefore, the m^6^A-regulated expression of RRM2B should be down to the positive effects of m^6^A in the mRNA stability of *RRM2B* mRNA.

Regarding the translation efficiency of the endogenous RRM2B mRNA, which is defined as the ratio of protein production (RRM2B/GAPDH) to mRNA abundance [35], knock-down of METTL3 had no effect on the translation efficiency of RRM2B in RKO cells (Fig. S3I). Further, polysome profiling analysis (Fig. S3J) confirmed that the relative ribosome occupancy of RRM2B in monosome and polysome fractions (levels of RRM2B mRNA/total RNA) were comparable between sh-control and sh-*METTL3* RKO cells (Fig. S3K). To assess whether m6A can post-translationally regulate the expression of RRM2B, both sh-control and sh-*METTL3* RKO cells were further treated with cycloheximide (CHX) to inhibit translation. Our data revealed that the half-life of RRM2B protein had no significant difference between sh-Control and *sh-METTL3* cells (Fig. S3L). All these data indicated that METTL3 positively regulates the mRNA stability of *RRM2B*, without affecting its transcription, nuclear export, translation efficiency or protein stability.

### m^6^A stabilized *RRM2B* mRNA via methylation of A1240 at 3′UTR

**Figure 4.**
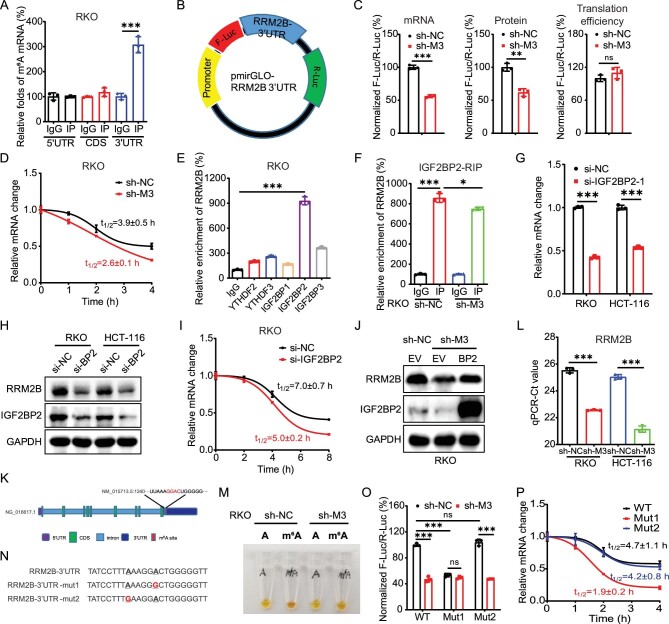
m^6^A stabilized *RRM2B* mRNA via methylation of A1240 at 3′UTR. (A) The m^6^A in 5′UTR, CDS or 3′UTR of *RRM2B* in RKO cells were analysed via m^6^A-RIP–qPCR using fragmented RNA. (B) Schematic representation of pmirGLO-*RRM2B* 3′UTR reporter. (C) The mRNA abundance, luciferase activity and translation efficiency of F-Luc in sh-control and sh-*METTL3* RKO cells transfected with pmirGLO-*RRM2B* 3′UTR reporter for 24 h. (D) After treatment with Act-D for the indicated times, the mRNA levels of *F-Luc* were checked in sh-control and sh-*METTL3* RKO cells transfected with pmirGLO-*RRM2B* 3′UTR reporter for 24 h. (E) RIP–qPCR analysis of *RRM2B* mRNA in RKO cells by use of antibody of YTHDF2, YTHDF3 and IGF2BP1-3. (F) IGF2BP2 RIP–qPCR analysis of *RRM2B* mRNA in sh-control and sh-*METTL3* RKO cells. (G and H) The mRNA (G) and protein (H) expression of RRM2B in RKO and HCT-116 cells transfected with si-NC or si-IGF2BP2 for 24 h. (I) After treatment with Act-D for the indicated times, the mRNA levels of *RRM2B* were checked in RKO cells transfected with si-NC or si-IGF2BP2 for 24 h. (J) The protein expression of RRM2B in sh-control and sh-*METTL3* RKO cells transfected with vector control or IGF2BP2 plasmid for 24 h. (K) Schematic representation of positions of m^6^A motifs within *RRM2B* mRNA. (L) The threshold cycle (Ct) of qPCR showing SELECT results for detecting m^6^A site in the potential m^6^A site of RRM2B in sh-control and sh-*METTL3* RKO and HC-T116 cells. (M) m^6^A-Rol-LAMP products of methylation modified site (A1240) and negative site (A1235) of *RRM2B* mRNA in sh-control or sh-*METTL3* RKO cells were detected using SYBR Green I (the trade name of a DNA dye). (N) Schematic representation of mutation in 3′UTR to investigate the m^6^A roles on RRM2B expression. (O) The relative luciferase activity of F-Luc/R-Luc of pmirGLO-*RRM2B* 3′UTR WT, Mut1 and Mut2 reporter in sh-control and sh-*METTL3* RKO cells. (P) After treatment with Act-D for the indicated times, the mRNA levels of *F-Luc* were checked in sh-control and sh-*METTL3* RKO cells transfected with pmirGLO-*RRM2B* 3′UTR WT, Mut1 and Mut2 reporter for 24 h. Data are presented as mean ± SD from three independent experiments. *P < 0.05; **P < 0.01; ***P < 0.001; ns, no significant, using Student's *t*-test between two groups and using one-way ANOVA followed by Bonferroni test for multiple comparisons.

We further investigated the methylation site and reader protein responsible for m^6^A-stablized *RRM2B* mRNA. m^6^A-RIP–PCR using fragmented poly^+^ RNA indicated that the relative enrichment of 3′UTR of *RRM2B* mRNA was much greater than that of coding sequence (CDS) in RKO cells (Fig. 4A), which is consistent with m^6^A-seq results (Fig. 3G). To investigate whether m^6^A-methylated 3′UTR was involved in the m^6^A-regulated mRNA stability of *RRM2B* mRNA, we constructed 3′UTR reporters containing wild-type *RRM2B* 3′UTR behind the firefly luciferase reporter gene by use of the pmiR-GLO vector (Fig. 4B). The luciferase assay illustrated that the levels of F-Luc in sh-*METTL3* RKO cells were significantly decreased, which was due to the downregulation of *F-Luc* mRNA and not the translation efficiency (Fig. 4C). Further, the addition of *RRM2B* 3′UTR decreased the half-life of *F-Luc* mRNA in sh-*METTL3* RKO cells (Fig. 4D). It further confirmed that m^6^A-methylated 3′UTR mediated the METTL3-regulated mRNA stability of *RRM2B*.

The m^6^A binding proteins insulin-like growth factor 2 mRNA-binding proteins (IGF2BPs; including IGF2BP1/2/3), but not YTH-domain family proteins YTHDF2/3, recognize and stabilize m^6^A-modified cellular RNAs [36]. RIP–qPCR showed that IGF2BP2 (not IGF2BP1/3 or YTHDF2/3) can bind with *RRM2B* mRNA in RKO cells (Fig. 4E). Further, the binding between IGF2BP2 and *RRM2B* mRNA was decreased in sh-*METTL3* RKO cells (Fig. 4F). Knock-down of IGF2BP2 was able to suppress the mRNA (Fig. 4G) and protein (Fig. 4H) expression of RRM2B in both RKO and HCT-116 cells. It was due to that knock-down of IGF2BP2 that there was suppression of the mRNA stability of RRM2B (Fig. 4I). Furthermore, overexpression of IGF2BP2 reversed the knock-down of METTL3-suppressed mRNA (Fig. S4A) and protein (Fig. 4J) expression of RRM2B. All these data suggested that IGF2BP2 mediated m^6^A-regulated expression of RRM2B.

We further investigated the methylation site for m^6^A-regulated mRNA stability of RRM2B. One GGAC motif with A1240 in *RRM2B* 3′UTR was identified (Fig. 4K), which was consistent with the positions and numbers of peaks identified by using m^6^A RIP-seq. In order to confirm the methylation of **A1240** within *RRM2B* 3′UTR, its position was checked using the ‘SELECT′ method [37] in RKO and HCT-116 cells. SELECT showed that knock-down of METTL3 decreased the methylation levels of A1240 (Fig. 4L) while the nearby nucleotide A1235 without m^6^A modification had a significantly lower Ct value than that of A1240 (Fig. S4B). The methylation of A1240 was further confirmed using our recently developed m^6^A-Rol-LAMP based on rolling circle amplification and loop-mediated isothermal amplification (LAMP) (Fig. 4M and Fig. S4C).

We further mutated A1240 within *RRM2B* 3′UTR to investigate its roles in the m^6^A-regulated mRNA stability of RRM2B (Fig. 4N). The mutation of A1240, while not at the control site (A1235) in 3′UTR, resulted in a downregulation of luciferase activity of F-Luc (Fig. 4O) whereas the mutation-induced downregulation of luciferase activity of F-Luc was reversed in sh-*METTL3* cells. Further, the mutation of 3′UTR A1240 decreased the mRNA stability of F-Luc (Fig. 4P). All data confirmed that A1240 within *RRM2B* 3′UTR mediated m^6^A-regulated mRNA stability of *RRM2B*.

### m^6^A-stablized *OPA1* mRNA regulated mitochondrial fusion

**Figure 5.**
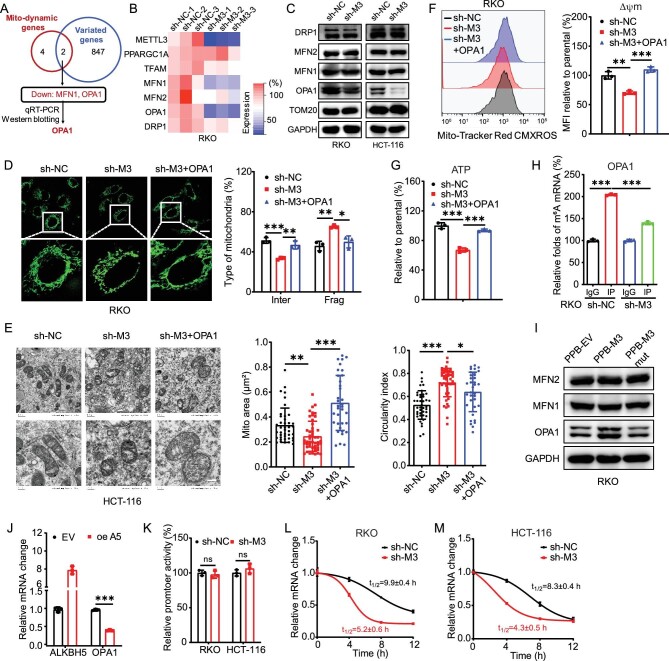
m^6^A-stablized *OPA1* mRNA regulated mitochondrial fusion. (A) The analysis process diagram to identify OPA1 as METTL3-regulated targets that were involved in mitochondrial dynamics in CRC cells. (B) The mRNA expression of *PGC-1α, TFAM, Mfn1, Mfn2, OPA1* and *Drp1* in sh-control and sh-*METTL3* RKO cells. (C) The protein expression of TOM20, MFN1, MFN2, OPA1 and DRP1 in sh-control and sh-*METTL3* CRC cells. (D) Representative confocal images of the mitochondrial morphology in sh-control and sh-*METTL3* RKO cells transfected with vector control, OPA1 plasmid for 24 h (scale bar, 20 μm). (E) Representative transmission electron microscopy images of the mitochondria in sh-control, sh-*METTL3* and OPA1 stable overexpression sh-*METTL3* HCT116 cells. The average mitochondrial area and circularity indexes were quantified via TEM analysis (scale bar, 200 nm). (F and G) Mitochondrial membrane potential (F) and intracellular ATP levels (G) in sh-control and sh-*METTL3* RKO cells transfected with vector control, OPA1 plasmid for 24 h (scale bar, 20 μm). (H) m^6^A RIP–qPCR analysis of *OPA1* in sh-control and sh-*METTL3* RKO cells. (I) The protein expression of OPA1, MFN1 and MFN2 in RKO cells transfected with vector control, METTL3 WT plasmid or METTL3 DA mutant plasmid for 24 h. (J) The mRNA levels of *OPA1* in RKO cells transfected with vector control, ALKBH5 plasmid for 24 h. (K) Cells were transfected with pGL3-Basic-*OPA1*-luc reporter and pRL-TK plasmid for 24 h. The promoter activities were presented as the ratios between the reporter and pRL-TK plasmid. (L and M) After treatment with Act-D for the indicated times, the mature mRNA levels of OPA1 were checked in sh-control and sh-*METTL3* RKO (L) or HCT-116 (M) cells. Data are presented as mean ± SD from three independent experiments. *P < 0.05; **P < 0.01; ***P < 0.001; ns, no significant, using Student's *t*-test between two groups and using one-way ANOVA followed by Bonferroni test for multiple comparisons.

Previous studies indicated that key proteins involved in mitochondrial dynamics such as mitochondrial biogenesis (PPARGC1A, TFAM), fusion (MFN1, MFN2, OPA1) and fission (DRP1) may be m^6^A-methylated [1,38]. We further investigated whether these key proteins (PPARGC1A, TFAM, MFN1, MFN2, OPA1 and DRP1) participate in m^6^A-regulated mitochondrial fusion. Overlap analysis showed that two candidates (down: MFN1 and OPA1) overlap among the key proteins and variated genes in sh-*METTL3* RKO cells (Fig. 5A). qRT–PCR showed that consistent knock-down of METTL3 decreased the mRNA expression of MFN1 and OPA1 in both RKO (Fig. 5B) and HCT-116 (Fig. S5A) cells. Western blot analysis showed that knock-down of METTL3 only decreased the protein expression of OPA1, while it had no effect on MFN1, in both RKO and HCT-116 cells (Fig. 5C).

Although the promotional roles of OPA1 in mitochondria have been well illustrated [39], we further checked its roles in m^6^A-regulated mitochondria fusion of CRC cells. Results showed that overexpression of OPA1 (Fig. S2B) can reverse the reduced mitochondria fusion (Fig. 5D and E), membrane potential (Fig. 5F) and ATP generation (Fig. 5G) of sh-*METTL3* RKO cells. This confirmed the essential role of OPA1 in m^6^A-regulated mitochondria fusion of CRC cells.

OPA1 is cleaved by mitochondrial peptidases OMA1 and YME1L in a stress-dependent manner [40]. Knock-down of METTL3 had no significant effect on the mRNA expression of either OMA1 or YME1L in RKO cells (Fig. S5B). We further investigated whether m^6^A regulated the expression of OPA1 via directly methylating its mRNA. m^6^A-seq data confirmed that mRNA of OPA1 was m^6^A-methylated (Fig. S5C). m^6^A-RIP–qPCR confirmed a 2-fold m^6^A antibody-enriched *OPA1* mRNA in RKO cells, while this enrichment significantly decreased in sh-*METTL3* RKO cells (Fig. 5H). Similar results were also observed in HCT-116 cells (Fig. S5D). This indicated that *OPA1* mRNA was m^6^A-methylated.

Further, the transfections of WT METTL3, but not catalytically inactive METTL3 mutant DA (D395A), increased the expression of OPA1 in RKO cells (Fig. 5I) while neither WT-METTL3 nor inactive METTL3 mutant had an effect on the protein expression of MFN1 or MFN2 in RKO cells (Fig. 5I). Further, overexpression of ALKBH5 decreased the mRNA (Fig. 5J) and protein (Fig. S5E) expression of OPA1 in RKO cells. This indicated that METTL3 can positively regulate the expression of OPA1 in CRC cells in an m^6^A-dependent manner.

We further investigated the mechanisms for m^6^A-regulated expression of OPA1. Similarly to RRM2B, the luciferase reporter assay using pGL3-Basic-*OPA1*-luc showed that there was no significant difference in the luciferase activity of the *OPA1* promoter between sh-control and sh-*METTL3* cells (Fig. 5K). qRT–PCR analysis consistently showed that the levels of precursor mRNA of *OPA1* were comparable between sh-control and sh-METTL3 cells (Fig. S5F). This indicated that m^6^A had no significant effect on the transcription of OPA1. Similarly, the subcellular localization of *OPA1* mRNA (Fig. S5G) and half-life of OPA1 protein (Fig. S5H) in sh-control and sh-*METTL3* cells were comparable. In addition, knock-down of METTL3 had no effect on the translation efficiency of endogenous *OPA1* mRNA in RKO cells (Fig. S5I). Further, ribosome profiling analysis confirmed that there is no significant difference between the ribosome occupancy of *OPA1* in monosome and polysome fractions of sh-control and sh-*METTL3* RKO cells (Fig. S5J). We then tested its effect on mRNA stability. The results showed that knock-down of METTL3 significantly decreased the mRNA stability of *OPA1* in both RKO (Fig. 5L) and HCT-116 (Fig. 5M) cells. All these data indicated that METTL3 positively regulated the mRNA stability of *OPA1*, while it had no effect on its transcription, nuclear export, translation efficiency or protein stability.

### m^6^A methylation at A2212 stabilized *OPA1* mRNA via binding with IGF2BP2

**Figure 6.**
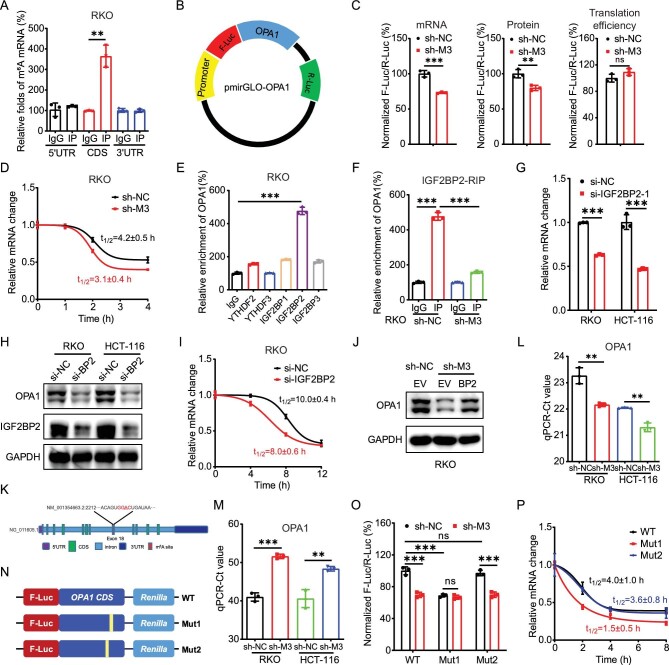
m^6^A methylation at A2212 stabilized *OPA1* mRNA via binding with IGF2BP2. (A) The m^6^A in 5′UTR, CDS or 3′UTR of *RRM2B* in RKO cells were analysed via m^6^A-RIP–qPCR using fragmented RNA. (B) Schematic representation of pmirGLO-*OPA1* CDS reporter. (C) The mRNA abundance, luciferase activity and translation efficiency of F-Luc in sh-control and sh-*METTL3* RKO cells transfected with pmirGLO-*OPA1* CDS reporter for 24 h. (D) After treatment with Act-D for the indicated times, the mRNA levels of *F-Luc* were checked in sh-control and sh-*METTL3* RKO cells transfected with pmirGLO-OPA1 CDS reporter for 24 h. (E) RIP–qPCR analysis of *OPA1* mRNA in RKO cells by use of antibody of YTHDF2, YTHDF3 and IGF2BP1∼3. (F) IGF2BP2 RIP–qPCR analysis of *OPA1* mRNA in sh-control and sh-*METTL3* RKO cells. (G and H) The mRNA (G) and protein (H) expression of OPA1 in RKO and HCT-116 cells transfected with si-NC or si-IGF2BP2 for 24 h. (I) After treatment with Act-D for the indicated times, the mRNA levels of *OPA1* were checked in RKO cells transfected with si-NC or si-IGF2BP2 for 24 h. (J) The protein expression of OPA1 in sh-control and sh-*METTL3* RKO cells transfected with vector control or ALKBH5 plasmid for 24 h. (K) Schematic representation of positions of m^6^A motifs within *OPA1* mRNA. (L) The threshold cycle (Ct) of qPCR showing SELECT results for detecting m^6^A site in the potential m^6^A site of *OPA1* in sh-control and sh-*METTL3* RKO and HCT116 cells. (M) m^6^A-Rol-LAMP products of methylation modified sites (A2212) of *OPA1* mRNA in sh-control or sh-*METTL3* RKO and HC-T116 cells were detected using SYBR Green I. (N) Schematic representation of mutation in CDS to investigate the m^6^A roles on OPA1 expression. (O) The relative luciferase activity of F-Luc/R-Luc of pmirGLO-*OPA1* CDS WT, Mut1 and Mut2 reporter in sh-control and sh-*METTL3* RKO cells. (P) After treatment with Act-D for the indicated times, the mRNA levels of *F-Luc* were checked in sh-control and sh-*METTL3* RKO cells transfected with pmirGLO-*OPA1* CDS WT, Mut1 and Mut2 reporter for 24 h. Data are presented as mean ± SD from three independent experiments. *P < 0.05; **P < 0.01; ***P < 0.001; ns, no significant, using Student's *t*-test between two groups and using one-way ANOVA followed by Bonferroni test for multiple comparisons.

We further investigated the methylation site and reader protein responsible for m^6^A-stablized *OPA1* mRNA. m^6^A-RIP–PCR using fragmented poly + RNA demonstrated a significantly greater relative enrichment of the CDS of *OPA1* mRNA compared with the 5′UTR in RKO cells (Fig. 6A), which was consistent with the prediction results of *OPA1* mRNA by use of the m^6^A sites predictor SRAMP (http://www.cuilab.cn/sramp) (Fig. S6A). To investigate whether m^6^A-methylated CDS were involved in m^6^A-regulated stability of *OPA1* mRNA, we constructed CDS reporters by inserting the wild-type OPA1 CDS downstream of the firefly luciferase reporter gene by use of the pmiR-GLO vector (Fig. 6B). The luciferase assay revealed a significant decrease in the luciferase expression of F-Luc in sh-*METTL3* RKO cells, which was attributed to the downregulation of *F-Luc* mRNA rather than a decrease in the translation efficiency (Fig. 6C). Further, the addition of *OPA1* CDS decreased the half-life of *F-Luc* mRNA in sh-*METTL3* RKO cells (Fig. 6D). This further confirmed that m^6^A-methylated CDS mediates the METTL3-regulated mRNA stability of *OPA1*.

RIP–qPCR showed that IGF2BP2, and not IGF2BP1/3 or YTHDF2/3, can bind with *OPA1* mRNA (Fig. 6E). Further, the binding between IGF2BP2 and *OPA1* mRNA was decreased in sh-*METTL3* RKO cells (Fig. 6F). Knock-down of IGF2BP2 suppressed the mRNA (Fig. 6G) and protein (Fig. 6H) expression of OPA1 in both RKO and HCT-116 cells. That knock-down of IGF2BP2 decreased the mRNA stability of OPA1 (Fig. 6I). Furthermore, overexpression of IGF2BP2 can reverse the knock-down of METTL3-suppressed mRNA (Fig. S6B) and protein (Fig. 6J) expression of OPA1. All data suggested that IGF2BP2 mediated m^6^A-regulated expression of OPA1.

As shown in Fig. 6K, one GGAC motif at exon 18 in *OPA1* CDS was identified, which is consistent with the positions and numbers of peaks identified using the m^6^A sites predictor (Fig. S6A). The m^6^A methylation of **A2212** within the GGAC motif at exon 18 in *OPA1* CDS was confirmed using the ‘SELECT’ method in RKO and HCT-116 cells, whilst knock-down of METTL3 can decrease the methylation levels of A2212 (Fig. 6L), whereas the nearby nucleotide A2207 without m^6^A modification had a significantly lower Ct value than that of A2212 (Fig. S6C). The methylation of A2212 was further confirmed using our recently developed m^6^A-Rol-LAMP assay (Fig. 6M and Fig. S6D).

We further mutated A2212 within *OPA1* CDS to investigate its roles in the m^6^A-regulated mRNA stability of *OPA1* (Fig. 6N and Fig. S6E). The mutation of A2212 but not the control site (A2207) in CDS resulted in a decrease in F-Luc activity (Fig. 6O), which was abolished in sh-*METTL3* cells. Further, the mutation of CDS A2212 decreased the mRNA stability of *F-Luc* (Fig. 6P). All these data confirmed that the methylation of **A2212** within *OPA1* CDS was involved in the m^6^A-regulated mRNA stability of OPA1.

### Generally or specifically targeting m^6^A suppressed mitochondria fusion

**Figure 7.**
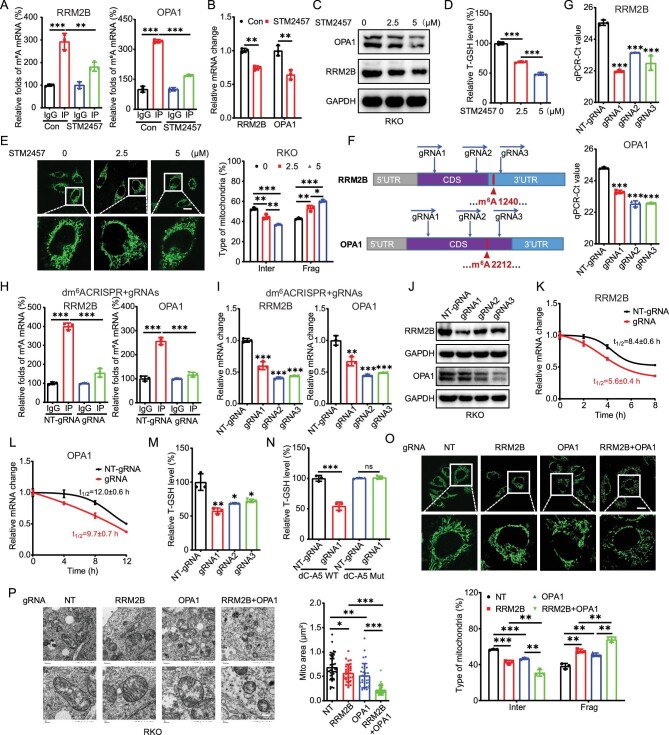
Generally or specifically targeting m^6^A-regulated mitochondria fusion. (A) m^6^A RIP–qPCR analysis of *RRM2B* or *OPA1* in RKO cells treated with METTL3 inhibitor STM2457 for 48 h. (B and C) The mRNA (B) or protein (C) levels of RRM2B or OPA1 in RKO cells treated with STM2457 for 48 h. (D) The T-GSH levels in RKO cells treated with STM2457 for 48 h. (E) Representative confocal images of the mitochondrial morphology in RKO cells treated with STM2457 for 48 h. (F) Schematic representation of positions of m^6^A site within *RRM2B* or *OPA1* mRNA and the regions targeted by three gRNAs, respectively. (G) The threshold cycle of qPCR showing SELECT results for detecting m^6^A site in *RRM2B* or *OPA1* in RKO cells transfected with dCas13b-ALKBH5 combined with gRNA negative control or gRNA1/2/3, respectively, for 24 h. (H) m^6^A-RIP–PCR analysis of *RRM2B* or *OPA1* mRNA in RKO cells transfected with dCas13b-ALKBH5 combined with gRNA negative control or gRNA1/2/3, respectively, for 24 h. (I and J) The mRNA (I) and protein (J) levels of *RRM2B* or *OPA1* in RKO cells transfected with dCas13b-ALKBH5 combined with gRNA negative control or gRNA1/2/3, respectively, for 24 h. (K and L) RKO cells were transfected with gRNA negative control, dCas13b-ALKBH5, with gRNA1 for *RRM2B* or gRNA3 for *OPA1* for 24 h and then further treated with Act-D for the indicated times. The mRNA level of *RRM2B* (K) and *OPA1* (L) was checked using qRT–PCR. (M) The T-GSH levels in RKO cells transfected with gRNA negative control, gRNA1/2/3 for *RRM2B* and dCas13b-ALKBH5 for 24 h. (N) The T-GSH levels in RKO cells transfected with gRNA negative control, gRNA1 for *RRM2B* and dCas13b-ALKBH5 or dCas13b-ALKBH5-Mut for 24 h. (O) The representative confocal images of the mitochondrial morphology and the percentages of mitochondrial types in RKO cells transfected with gRNA negative control, gRNA1 for *RRM2B* or gRNA3 for *OPA1* and dCas13b-ALKBH5 for 24 h (scale bar, 20 μm). (P) Representative transmission electron microscopy images of the mitochondria in RKO cells transfected with gRNA negative control, gRNA1 for *RRM2B* or/and gRNA3 for *OPA1* and dCas13b-ALKBH5; the average mitochondrial area was quantified via TEM analysis (scale bar, 200 nm). Data are presented as mean ± SD from three independent experiments. *P < 0.05; **P < 0.01; ***P < 0.001; ns, no significant, using Student's *t*-test between two groups and using one-way ANOVA followed by Bonferroni test for multiple comparisons.

Firstly, we evaluated the potential roles of STM2457, a highly potent and selective inhibitor of METTL3/14 [41], on mitochondria dynamics and expression of RRM2B and OPA1. Results showed that STM2457 significantly decreased the m^6^A enrichment of *RRM2B* and *OPA1* in RKO cells (Fig. 7A). Further, STM2457 suppressed the mRNA (Fig. 7B) and protein (Fig. 7C) expression of RRM2B and OPA1 in RKO cells. In addition, STM2457 decreased the levels of T-GSH in RKO cells via a concentration-dependent manner (Fig. 7D). Staining with MitoTracker Green showed that treatment with STM2457 resulted in smaller and shorter rounded mitochondria (Fig. 7E). It confirmed that targeting m^6^A via the METTL3 inhibitor can suppress mitochondria fusion of CRC cells.

We further specifically demethylated m^6^A of *RRM2B* and *OPA1* via development of the dm^6^ACRISPR, which fuses the catalytically inactive Type VI-B Cas13 enzyme with the m^6^A demethylase ALKBH5, as previously described [42] (Fig. S7A). Three gRNAs were designed to target the 3′UTR of *RRM2B* mRNA or the CDS of *OPA1* mRNA, respectively (Fig. 7F). Our data showed that co-transfection of wild-type Cas13b with three gRNAs can significantly reduce the mRNA levels of RRM2B and OPA1 (Fig. S7B), indicating that all gRNAs functioned efficiently.

SELECT–qPCR analysis showed a significant decrease in m^6^A levels at the targeted sites of RRM2B and OPA1 in RKO cells following transfection with gRNAs and dCas13b-ALKBH5 (Fig. 7G). Additionally, m^6^A-RIP–PCR confirmed that gRNAs and dCas13b-ALKBH5 can effectively decrease the m^6^A methylation of *RRM2B* and *OPA1* in RKO cells (Fig. 7H). Results showed that dm^6^ACRISPR targeting *RRM2B* or *OPA1* led to a significant downregulation of mRNA (Fig. 7I) and protein (Fig. 7J) expression of RRM2B and OPA1 in RKO cells. It was due to dm^6^ACRISPR with specific gRNA that there was a decrease in the mRNA stability of *RRM2B* (Fig. 7K) and *OPA1* (Fig. 7L) in RKO cells.

We further investigated whether dm^6^ACRISPR targeting *RRM2B* and *OPA1* can modulate mitochondria fusion. Our data showed that dm^6^ACRISPR targeting *RRM2B* significantly decreases the levels of T-GSH in RKO cells (Fig. 7M). However, dm^6^ACRISPR targeting OPA1 had no similar effects on the levels of T-GSH (Fig. S7C), but it affected GSH redox change (Fig. S7D). In addition, gRNA1 for *RRM2B* combined with dCas13b-ALKBH5 mutant did not produce a significant effect (Fig. 7N). Further, dm^6^ACRISPR targeting *RRM2B* and *OPA1*, respectively, resulted in smaller and shorter rounded mitochondria in RKO cells (Fig. 7O and P). The combination of gRNA for *RRM2B* and *OPA1* showed a synergistic effect on mitochondria fusion (Fig. 7O and P). These data suggested that generally or specifically targeting m^6^A can regulate mitochondria fusion in CRC cells.

### Oncogenic roles of m^6^A-regulated mitochondria dynamics in CRC development

**Figure 8.**
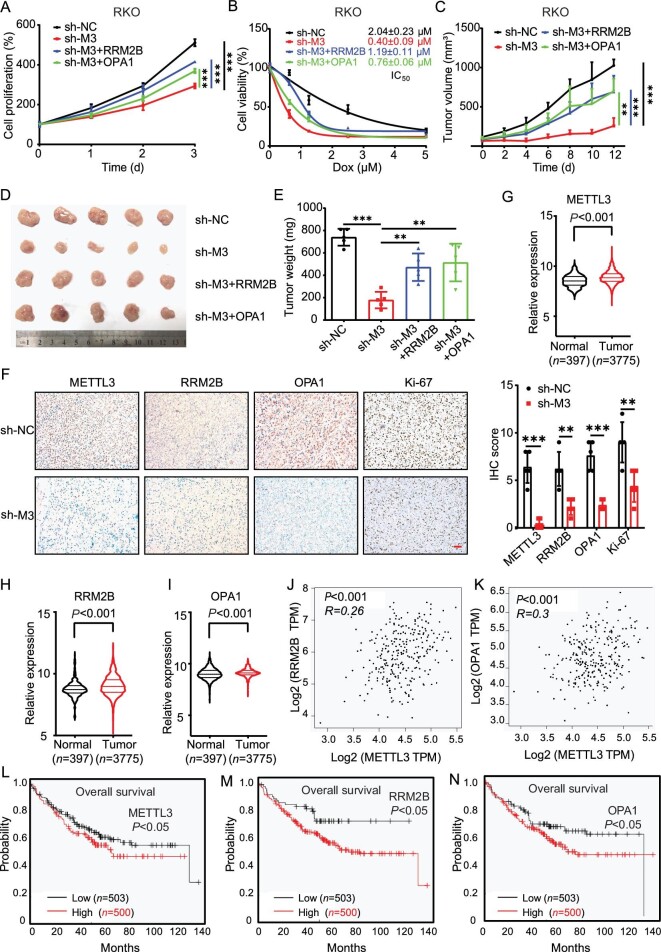
Oncogenic roles of m^6^A-regulated mitochondria dynamics in CRC development. (A) The relative cell proliferation of sh-control, sh-*METTL3* and RRM2B or OPA1 stable overexpression sh-*METTL3* RKO cells. (B) sh-control, sh-*METTL3* and RRM2B or OPA1 stable overexpression sh-*METTL3* RKO cells were treated with increasing concentrations of Dox for 24 h and the cell proliferation was tested. (C) The tumor growth curves of sh-control, sh-*METTL3* and RRM2B or OPA1 stable overexpression sh-*METTL3* RKO xenografts. (D and E) The tumor volume (D) and tumor weight (E) of sh-control, sh-*METTL3* and RRM2B or OPA1 stable overexpression sh-*METTL3* RKO xenografts at the end of the experiment. (F) IHC (METTL3, RRM2B, OPA1 and Ki67)-stained paraffin-embedded sections obtained from sh-control, sh-*METTL3* RKO cells. The scale bar is 50 μm. (G–I) Expression of METTL3 (G), RRM2B (H) or OPA1 (I) in CRC tumor tissues and adjacent normal mucosa tissues from GENT database. (J and K) Correlation between METTL3 and RRM2B (J) or OPA1 (K) in CRC patients from Gene Expression Profiling Interactive Analysis (GEPIA) database. (L–N) The Kaplan–Meier survival curves of OS based on METTL3 (L), RRM2B (M) or OPA1 (N) in CRC cancer patients from The Cancer Genome Atlas (TCGA) database. Data are presented as mean ± SD from three independent experiments. *P < 0.05; **P < 0.01; ***P < 0.001; ns, no significant, using Student's *t*-test between two groups and using one-way ANOVA followed by Bonferroni test for multiple comparisons.

We further checked the *in vivo* effect of m^6^A-regulated mitochondria dynamics in CRC progression. Cell viability assay showed that overexpression of RRM2B and OPA1 can reverse the suppressed growth effect of sh-*METTL3* RKO cells (Fig. 8A). Further, dCas13b-ALKBH5 with gRNAs of RRM2B or OPA1 decreased the proliferation in both RKO (Fig. S7E) and HCT-116 (Fig. S7F) cells. Furthermore, our data suggested that knock-down of METTL3 enhanced the sensitivity of RKO cells to doxorubicin (Dox), yet overexpression of RRM2B and OPA1 was able to mitigate these effects and decrease Dox sensitivity (Fig. 8B). This suggested that mitochondria dynamics was involved in METTL3-regulated growth and chemosensitivity of CRC cells.

RKO sh-control, sh-*METTL3* and RRM2B or OPA1 stable overexpression in sh-*METTL3* cells was used to establish xenografts. Results showed that knock-down of METTL3 significantly inhibited the growth of RKO xenografts, while the overexpression of RRM2B or OPA1 can rescue this effect (Fig. 8C). At the end of the experiment, the tumor volumes (Fig. 8D) and weights (Fig. 8E) in the sh-*METTL3* group were significantly lower than those measured in the sh-control group, and these effects were reversed by overexpression of RRM2B or OPA1. No significant change in body weight was observed among all the groups (Fig. S8A). Further, IHC showed that the expression of RRM2B, OPA1 and Ki67-positive staining, which recognizes a nuclear antigen expressed in proliferating cells, in the sh-*METTL3* group was decreased *in vivo* (Fig. 8F). This suggested that the knock-down of METTL3 can suppress the mitochondrial fusion and tumor growth of CRC cells.

At this point, we investigated the potential connection between m^6^A methylation, mitochondria dynamics and CRC development using clinical data from databases. The data form GENT indicated an upregulation in the expression of *METTL3* (Fig. 8G), *RRM2B* (Fig. 8H), *OPA1* (Fig. 8I) and *IGF2BP2* (Fig. S8B) in CRC tumor tissues compared with normal tissues. Furthermore, data from Gene Expression Profiling Interactive Analysis (GEPIA) demonstrated a positive correlation between METTL3 and RRM2B (Fig. 8J), as well as OPA1 (Fig. 8K) in CRC cancer patients. Consistently, the protein expression of METTL3 was positively correlated with the RRM2B (Fig. S8E) and OPA1 (Fig. S8F) in CRC cancer patients from the Clinical Proteomic Tumor Analysis Consortium (CPTAC) database. Moreover, the expression of IGF2BP2 exhibited a positive correlation with the RRM2B (Fig. S8C) and OPA1 (Fig. S8D) in CRC cancer patients. In addition, the expression of RRM2B (Fig. S8E) and OPA1 (Fig. S8F) was notably lower in Grade I CRC tissues compared with Grade II/III tissues. Further, CRC patients with increased expression of METTL3 (Fig. 8L), RRM2B (Fig. 8M), OPA1 (Fig. 8N) and IGF2BP2 (Fig. S8G) showed reduced overall survival (OS). Together, these data suggested that the m^6^A/mitochondria dynamics axis regulated CRC progression.

## DISCUSSION

**Figure 9.**
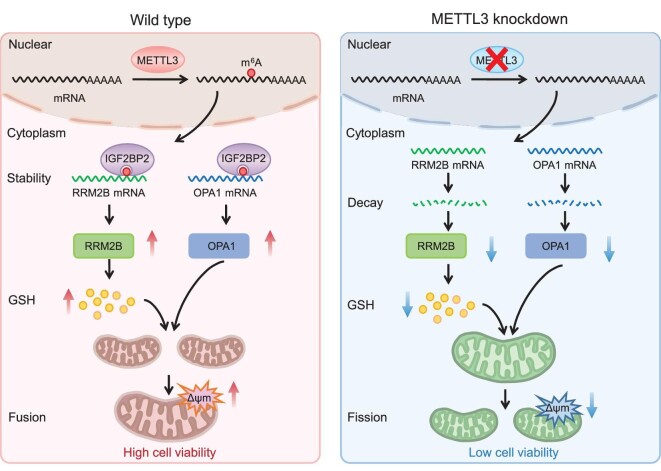
Working model of m^6^A-facilitated mitochondrial fusion of CRC cells via induction of GSH and stabilization of OPA1. In this model, METTL3 methylates the m^6^A modification of *RRM2B* and *OPA1* mRNAs, which in turn increases their mRNA stability by IGF2BP2, thereby increasing their protein expression, leading to promotion of GSH synthesis and mitochondrial fusion. Loss of METTL3 decreases the m^6^A levels of *RRM2B* and *OPA1* mRNAs, which are specifically recognized and stabilized by IGF2BP2, resulting in the decreased protein expression, thereby inhibiting GSH synthesis and mitochondrial fusion. The METTL3 knock-down cell with a depolarized mitochondrial membrane potential shows lower cell viability and higher sensitivity to antitumor drugs.

Dysregulated mitochondrial dynamics are critical for cancer progression such as metastasis, drug resistance and cancer stem cell survival [7,8]. Increasing evidence has confirmed that targeting mitochondrial dynamics and its regulatory factors is a potential therapeutic strategy for cancer therapy [10,43]. Our present study revealed that m^6^A methyltransferase accelerated mitochondria fusion of CRC cells via regulation of the RRM2B/GSH axis and induced the expression of OPA1 (Fig. 9). Mechanically, IGF2BP2 bound with the A1240 at 3′UTR of RRM2B and A2212 at exon 18 in OPA1 CDS increased their mRNA stability, respectively. General or specifically targeting m^6^A significantly suppressed mitochondria fusion of CRC cells. *In vivo* and clinical data confirmed the positive roles of m^6^A/mitochondrial dynamics in tumor growth and the progression of CRC cancer. Consistently, recent studies have indicated that m^6^A and METTL3 can promote CRC progression, such as by inducing growth and drug resistance, and inhibiting antitumor immunity [44–46]. Our results describe the potential roles of m^6^A in mitochondrial dynamics and also create the possibility of developing therapeutic strategies against CRC progression by targeting m^6^A/mitochondria pathways.

Recent studies indicate that m^6^A is critical for mitochondrial biogenesis and physiological functions [47]. For example, our previous study revealed that m^6^A can positively regulate the glycolysis of cancer cells via regulation of PDK4 [48]. Herein, we found that METTL3 can positively regulate the mitochondrial fusion via an m^6^A enzyme activity-dependent manner. One recent study consistently showed that the deletion of m^6^A demethylase FTO depletion induces mitochondrial fission in gastric cancer cells via caveolin-1 [20]. Further, FTO reduced the mRNA m^6^A of Drp1 and impaired the Drp1-mediated mitochondrial fragmentation [49]. Our data and previous studies confirmed the promotional effects of m^6^A on mitochondria dynamics and oncogenic roles of m^6^A/mitochondria in CRC development.

We identified that RRM2B/GSH is involved in m^6^A-regulated mitochondria fusion of CRC cells. Metabolomics analysis indicated that knock-down of METTL3 suppressed GSH levels in CRC cells, while the addition of GSH can reverse sh-*METTL3*-suppressed mitochondria fusion. RRM2B is a ribonucleotide reductase that protects glutathione synthetase (GSS) from proteasome degradation, thus maintaining the GSH concentration to prevent damage from lipid peroxide [50]. It participates in the regulation and modification of proteins and is also considered a vital component in tumor progression [51,52]. Since RRM2B is significantly upregulated in CRC tissues and associated with poor prognosis [53], our data suggest that m^6^A/RRM2B-regulated GSH generation and mitochondria fusion are potential targets for CRC therapy.

We further identified that m^6^A methylation of A2212 at exon 18 in OPA1 increase its mRNA stability and is involved in m^6^A-regualted mitochondria fusion of CRC cells. As the key regulator for cristae junction formation and respiratory chain super complexes, the IMM regulator OPA1 emerges as an intriguing candidate for targeted mitochondrial cancer therapy [54,55]. It is frequently amplified across pan-cancer genomic data sets and is associated with a poor prognosis and a heightened resistance to chemotherapy [56,57]. Our data revealed that METTL3 positively regulated the expression of OPA1, while overexpression of OPA1 reversed sh-*METTL3*-suppressed mitochondria fusion of CRC cells. Previous studies indicated mitochondrial peptidases OMA1 and YME1L cleaved OPA1 to regulate mitochondria dynamics [40]. Our present study revealed another layer of regulation factors for OPA1 expression and mitochondria fusion.

Developing inhibitors/activators of m^6^A-related proteins has become a hot spot in the field of anticancer epigenetic drugs [41]. Mitochondrial dysfunction and metabolic alterations have long been proposed to play a critical role in the pathogenesis of various cancers [7,58]. We therefore believe that novel, specific, effective and promising methods targeting m^6^A and mitochondrial dynamics will be developed, leading to a new generation in both cancer diagnosis and targeted therapy. Further, mitochondrial RNAs that have been discovered cover methylation, pseudouridylation and other modifications, which are involved in the biogenesis, stability and function of all mtRNA species [59,60]. Thus, the role of RNA modification in mtRNAs in mitochondria dynamics makes plenty of sense and remains to be further investigation.

Overall, our study sheds light on a novel relationship between mitochondria dynamics and m^6^A methylation. Specifically, m^6^A accelerated the mitochondria fusion of CRC cells via activation of the RRM2B/GSH axis and upregulation of OPA1. Given the numerous genes involved in mitochondrial dynamics, it is plausible that m^6^A modification indirectly regulates CRC mitochondrial dynamics by affecting other genes. Our study suggested that m^6^A regulates the mitochondrial dynamics of CRC cells, which has expanded our understanding of such interplays that are essential for therapeutic application.

## MATERIALS AND METHODS

### Mitochondrial morphology

To assess mitochondrial morphology, cells were seeded into confocal dishes and cultured with Mito-Tracker Green probe (Beyotime, China) and Hoechst 33342 staining solution (Beyotime, China) for 30 min at 37°C. They were then washed using phosphate buffer saline (PBS) three times for analysis using an Olympus Microscope (Olympus, Japan). Mitochondrial length was measured using ImageJ-MiNA [21] and categorized as elongated (˃3 μm), intermediate (0.5–3 μm) or fragmented (˂0.5 μm) types. The number of mitochondria in each category was counted from three independent experiments (>1000 mitochondria were analysed) and presented as mean of type percentage (± SD).

### EM

Following the indicated treatments, cells were dissociated, centrifuged and fixed in 4% glutaraldehyde (Solarbio, China) for 2 h at 4°C. They were then post-fixed in 1% osmium tetroxide for an additional hour at 4°C. The samples were dehydrated, embedded in epoxy resin and ultimately sectioned into 70-nm-thick slices for microscopic examination. These sections were post-stained using 5% uranyl acetate and examined under a transmission electron microscope. Images were randomly captured to measure the mitochondrial area and circularity using ImageJ software.

### Luciferase reporter assay

To assess the impact of m^6^A on the transcription of the target gene, we cloned the promoter region of the gene (–1000 to +100) into pGL3-Basic-Vector (Promega, USA) for promoter activity measurement. Cells were co-transfected with pGL3-basic-promoter and TK-Rluc reporter in a six-well plate for 24 h and the Dual-Luciferase Reporter Gene Assay Kit (Beyotime, China) was used to measure transcriptional activity. Renilla Luciferase (R-Luc) served as an internal control to normalize firefly luciferase (F-Luc) activity.

The CDS or 3′UTR sequence was subcloned into the dual-luciferase vector pmiGLO (Promega, USA). Mutagenesis of m^6^A sites (A to G) was conducted using a site-directed mutagenesis kit (Thermo Fisher, USA). The F-Luc activity values were normalized to the R-Luc activity values to reflect expression efficiency. The translation outcome was determined by comparing the F-Luc/R-Luc signal to mRNA abundance, while translation efficiency was defined as the quotient of reporter protein production divided by mRNA abundance[35]. All experiments were performed three times with consistent results.

### Statistical analyses

Data were presented as mean ± SD from a minimum of three independent experiments. Statistical analysis was performed using a two-tailed unpaired Student's *t*-test for comparing two groups, while one-way or two-way Analysis of Variance (ANOVA) followed by the Bonferroni test was used for multiple comparisons. All statistical tests were two-sided. Analysis was performed using SPSS 16.0 for Windows. A *P*-value of <0.05 was considered to be statistically significant. *P < 0.05, **P < 0.01, ***P < 0.001; ns, no significant.

## Supplementary Material

nwae039_Supplemental_Files
