# Supplementary material for: N6-methyladenosine facilitates mitochondrial fusion of colorectal cancer cells via induction of GSH synthesis and stabilization of OPA1 mRNA
Source: Natl Sci Rev. 2024 Jan 29;11(3):nwae039. doi: 10.1093/nsr/nwae039 (PMC10977914; doi:10.1093/nsr/nwae039)
Supplement: nwae039_Supplemental_Files [file nwae039_supplemental_files.zip › Tables/Table S5 Primers.docx]

**Table S5 Primers used in the present study**

| **Gene** | **Primer sequence** |
| --- | --- |
| **qRT-PCR** |  |
| RRM2B | forward: 5’- TTCTCAGGATCTTTTGCTGCTA -3’ |
|  | reverse: 5’- GACAGCATCAACAATGATCTCC -3’ |
| Pre-RRM2B | forward: 5’- GGCACAGGCTTCCTTCTGGA -3’ |
|  | reverse: 5’- AGGAACACTGCACTATAGGATGTGA -3’ |
| RRM1 | forward: 5’- CATCTCGAATCCAGAAGCTTTG -3’ |
|  | reverse: 5’- CAATGAGCTGTGATTATGCCAC-3’ |
| GPX3 | forward: 5’- CATAAGTGGCACCATTTACGAG -3’ |
|  | reverse: 5’- AAGGATCTCTGAGTTCTCTCCT -3’ |
| GAPDH | forward: 5’- GCACCGTCAAGGCTGAGAAC-3’ |
|  | reverse: 5’- TGGTGAAGACGCCAGTGGA -3’ |
| OPA1 | forward: 5’- TCTGCACACTCAGTTGAAGTAT-3’ |
|  | reverse: 5’- GCCTTTGTCATCTTTCTGCAAT-3’ |
| Pre-OPA1 | forward: 5’- CCGTTAATTCTGGGGCCTCTTGAG -3’ |
|  | reverse: 5’- GGCTCCTGTCATTCTGGGTCCTC -3’ |
| MFN1 | forward: 5’- GTGGCAAACAAAGTTTCATGTG-3’ |
|  | reverse: 5’- CACTAAGGCGTTTACTTCATCG-3’ |
| MFN2 | forward: 5’- GTGCTTCTCCCTCAACTATGAC-3’ |
|  | reverse: 5’- ATCCGAGAGAGAAATGGAACTC-3’ |
| DRP1 | forward: 5’- GAGATGGTGTTCAAGAACCAAC-3’ |
|  | reverse: 5’- CAATAACCTCACAATCTCGCTG-3’ |
| MFF | forward: 5’- ACTCGTAGGGCATACCAGCAGATC-3’ |
|  | reverse: 5’- GATTAGAAGTGGCGGCAGCAGAC-3’ |
| PPARGC1A | forward: 5’- CAGAGAGTATGAGAAGCGAGAG-3’ |
|  | reverse: 5’- AGCATCACAGGTATAACGGTAG-3’ |
| TFAM | forward: 5’-TTCCAAGAAGCTAAGGGTGATT-3’ |
|  | reverse: 5’- AGAAGATCCTTTCGTCCAACTT-3’ |
| METTL3 | forward: 5’- CTATCTCCTGGCACTCGCAAGA -3’ |
|  | reverse: 5’- GCTTGAACCGTGCAACCACATC-3’ |
| ALKBH5 | forward: 5’- CCAGCTATGCTTCAGATCGCCT -3’ |
|  | reverse: 5’- GGTTCTCTTCCTTGTCCATCTCC-3’ |
| HPRT | forward: 5’- TGACACTGGCAAAACAATGCA-3’ |
|  | reverse: 5’- GGTCCTTTTCACCAGCAAGCT-3’ |
| 18S rRNA | forward: 5’- CGGACAGGATTGACAGATTGATAGC-3’ |
|  | reverse: 5’- TGCCAGAGTCTCGTTCGTTATCG -3’ |
| Firefy-Luc | forward: 5’- GGTACTGTTGGTAAAGCCAC-3’ |
|  | reverse: 5’- CTCTTCATAGCCTTATGCAG-3’ |
| Renilla-Luc | forward: 5’- CAATGGGCAGGTGTCCACTC -3’ |
|  | reverse: 5’- GTTCTGGATCATAAACTTTC -3’ |
| **Mutation plasmid** | **Primer sequence** |
| RRM2B/3’UTR-Mut1 | forward: 5’- ATATCCTTTAAAGGCCTGGGGGTTTGCT -3’ |
|  | reverse: 5’- AGCAAACCCCCAGGCCTTTAAAGGATAT -3’ |
| RRM2B/3’UTR-Mut2 | forward: 5’- TATATCCTTTCAAGGACTGGG -3’ |
|  | reverse: 5’- CCCAGTCCTTGAAAGGATATA -3’ |
| OPA1/CDS-Mut1 | forward: 5’- TAAACAGTGGGCTGATAAACA-3’ |
|  | reverse: 5’- TGTTTATCAGCCCACTGTTTA -3’ |
| OPA1/CDS-Mut2 | forward: 5’- AAGCTTAAACAGTGGACTGAT-3’ |
|  | reverse: 5’- ATCAGTCCACTGTTTAAGCTT -3’ |
| **SELECT** | **Primer sequence** |
| RRM2B-X | forward: 5’- tagccagtaccgtagtgcgtg TTTTGAGCAAACCCCCAG -3’ |
|  | reverse: 5phos/ CCTTTAAAGGATATACT cagaggctgagtcgctgcat -3’ |
| RRM2B-N | forward: 5’- tagccagtaccgtagtgcgtg CAAACCCCCAGTCCTT -3’ |
|  | reverse: 5’- 5phos/ AAAGGATATACTTAAAA cagaggctgagtcgctgcat -3’ |
| OPA1-X | forward: 5’- tagccagtaccgtagtgcgtg TAGGAAGTTGTTTATCAG -3’ |
|  | reverse: 5phos/ CCACTGTTTAAGCTTG cagaggctgagtcgctgcat -3’ |
| OPA1-N | forward: 5’- tagccagtaccgtagtgcgtg GTTGTTTATCAGTCCAC -3’ |
|  | reverse: 5’- 5phos/ GTTTAAGCTTGATATC cagaggctgagtcgctgcat -3’ |
| **m^6^A-Rol-LAMP** | **Primer sequence** |
| rlProbe-RRM2B-m^6^A | 5’-5phos/cctttaaaggatatacTTTCGACACGACACGATTTGGAACTCTGCTCGACGGATTAAATAATACAGTCTGCCCACAACCTTTtgagcaaacccccag-3’ |
| rlProbe-RRM2B-A | 5’-5phos/atacttaaaatttttTTTCGACACGACACGATTTGGAACTCTGCTCGACGGATTAAATAATACAGTCTGCCCACAACCTTTcagtcctttaaagga-3’ |
| rlProbe-OPA1-m^6^A | 5’-5phos/ccactgtttaagcttgTTTCGACACGACACGATTTGGAACTCTGCTCGACGGATTAAATAATACAGTCTGCCCACAACCTTTggaagttgtttatcag-3’ |
| rlProbe-OPA1-A | 5’-5phos/aagcttgatatccactgTTTCGACACGACACGATTTGGAACTCTGCTCGACGGATTAAATAATACAGTCTGCCCACAACCTTTtatcagtccactgtt-3’ |
| **dm^6^ACRISPR** | **Primer sequence** |
| RRM2B-gRNA1 | 5’- AAAGCGCTCC ACCAAATTTT CATTTACAAT TCCATCACTG -3’ |
| RRM2B-gRNA2 | 5’- ATCCAAGTTC CACAAGTAAT CTGTCAGCTA CAAACTCAAT -3’ |
| RRM2B-gRNA3 | 5’- TGTCTGACCC CTCGCCCCAT GCTCTTAACC ACATAATGCC -3’ |
| OPA1-gRNA1 | 5’- GAATACACAG TATGATGGCA TTAGGATTCT GCATGTAAGC -3’ |
| OPA1-gRNA2 | 5’- ATTCAGTTTC AAGGTTAAAA CGTGTTGCTT TGAAACTATC -3’ |
| OPA1-gRNA3 | 5’- CAGATATGGA TCGGTCTTCC AAAGCATTGT GTTGAATAAC -3’ |
