# Supplementary material for: N6-methyladenosine facilitates mitochondrial fusion of colorectal cancer cells via induction of GSH synthesis and stabilization of OPA1 mRNA
Source: Natl Sci Rev. 2024 Jan 29;11(3):nwae039. doi: 10.1093/nsr/nwae039 (PMC10977914; doi:10.1093/nsr/nwae039)
Supplement: nwae039_Supplemental_Files [file nwae039_supplemental_files.zip › SI_NSR 20240123.docx]

**Supplementary data for**

**N6-methyladenosine facilitates mitochondrial fusion of colorectal cancer cells via induction of GSH synthesis and stabilization of *OPA1* mRNA**

**Zhou et al**

**
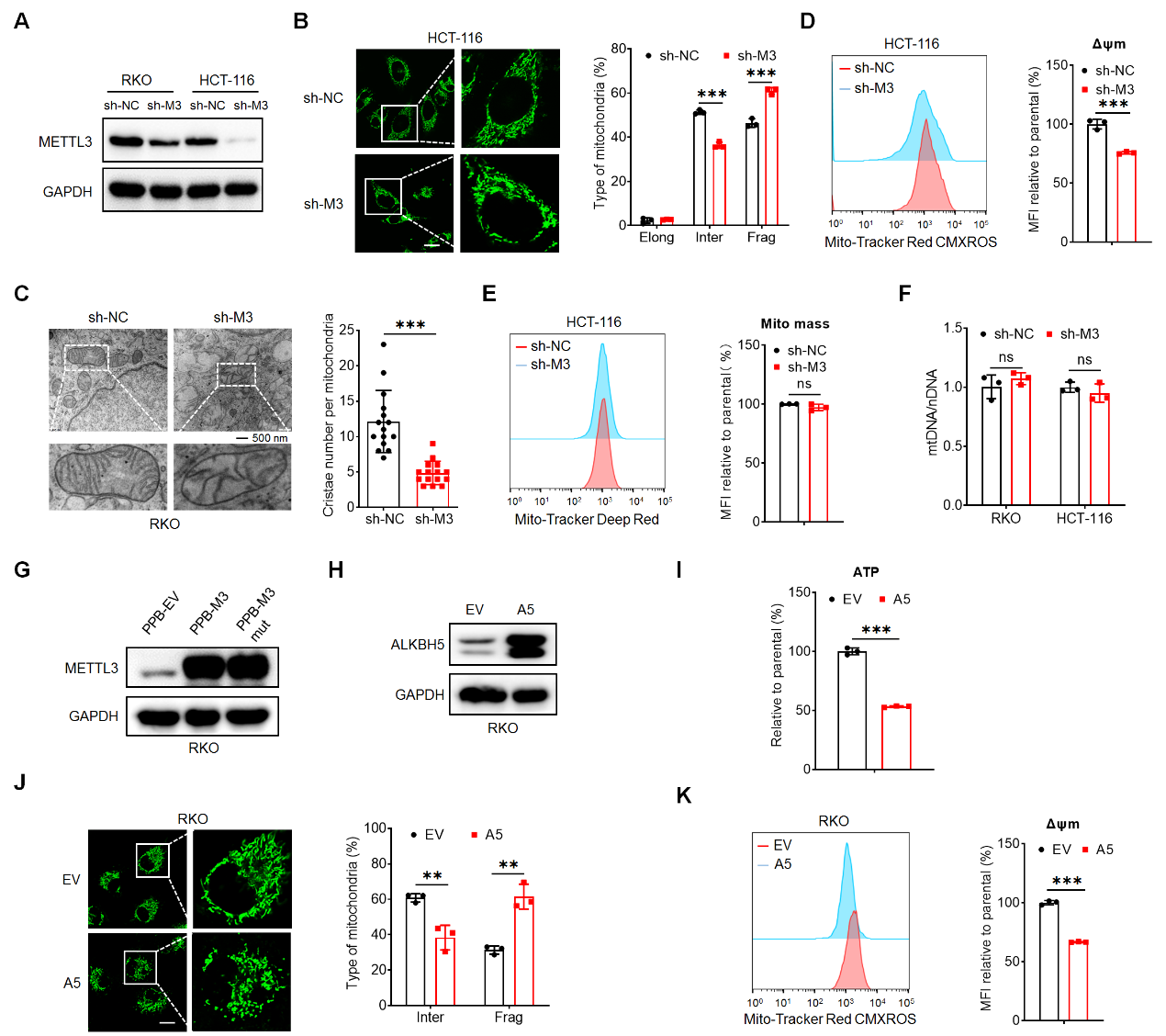
**

**Supplementary Figure 1 m^6^A facilitated mitochondria fusion of CRC cells**

1. The protein expression of METTL3 in sh-*METTL3* RKO, sh-*METTL3* HCT-116 cells and their corresponding control cells;
2. Representative confocal images of mitochondrial morphology in sh-control and sh-*METTL3* HCT-116 cells (left) and the percentages of mitochondrial types was measured (right);
3. Representative transmission electron microscopy images of the cristae morphology in sh-control and sh-*METTL3* RKO cells (scale bar, 500 nm); The cristae numbers per mitochondrial were quantified via TEM analysis
4. Mitochondrial membrane potential was measured in sh-control and sh-*METTL3* HCT-116 cells by flow cytometry (left) and Median fluorescence intensity was measured (right);
5. MitoTracker Deep Red staining was performed on sh-control and sh-*METTL3* HCT-116 cells to stain the mitochondria for flow cytometric analysis(left) and Median fluorescence intensity was measured (right);
6. RT-PCR was performed to quantify mitochondrial cytochrome B (cytoB as mtDNA) expression levels, which were normalized to β-actin expression levels in sh-control and sh-*METTL3* RKO or HCT-116 cells to assess the total mitochondrial mass;
7. The protein expression of METTL3 in RKO cells transfected with vector control, METTL3 WT plasmid, or METTL3 DA mutant plasmid for 24 h;
8. The protein expression of ALKBH5 in RKO cells transfected with vector control or ALKBH5 plasmid for 24 h;

(I~K) Intracellular ATP levels (I), representative confocal images of the mitochondrial morphology (J), and mitochondrial membrane potential (K) in RKO cells transfected with vector control or ALKBH5 plasmid for 24 h.

Data are presented as mean ± SD from three independent experiments. **p*<0.05, ***p*<0.01, ****p*<0.001, ns, no significant, by Student’s *t* test between two groups and by one-way ANOVA followed by Bonferroni test for multiple comparison.

**Related to Figure 1.**

**
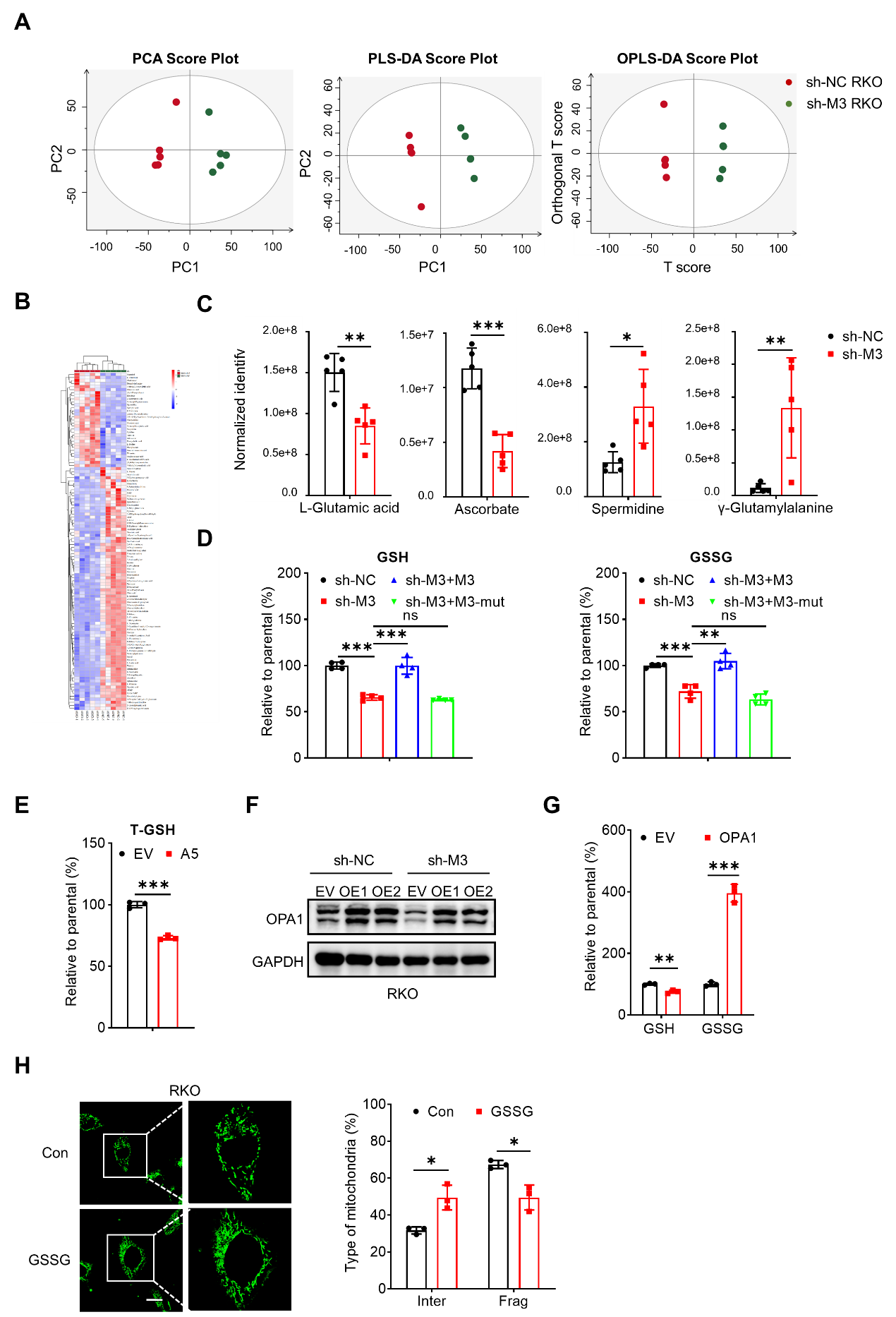
**

**Supplementary Figure 2 Glutathione was involved in m^6^A-induced mitochondrial fusion.**

1. Multivariate statistical analysis by PCA, PLS-DA and OPLS-DA of the metabolomics analysis in sh-control and sh-*METTL3* RKO cells;
2. The altered metabolites in sh-*METTL3* RKO cells compared to sh-control cells by metabolomics analysis;
3. The altered metabolites in the glutathione metabolism pathway of the metabolomics analysis;
4. The GSH and GSSG levels in sh-control and sh-*METTL3* RKO cells transfected with vector control, METTL3 WT plasmid, METTL3 DA mutant plasmid for 24 h;
5. The T-GSH levels in RKO cells transfected with vector control or ALKBH5 plasmid for 24 h;
6. The protein expression of OPA1 in RKO cells transfected with vector control or OPA1 plasmid for 24 h;
7. The GSH and GSSG levels in sh-*METTL3* RKO cells transfected with vector control or OPA1 plasmid for 24 h;
8. Representative confocal images of the mitochondrial morphology in sh-*METTL3* RKO cells treated with 100 μM GSSG for 24 h (scale bar, 20 μm);

Data are presented as mean ± SD from three independent experiments. **p*<0.05, ***p*<0.01, ****p*<0.001, by Student’s *t* test between two groups and by one-way ANOVA followed by Bonferroni test for multiple comparison.

**Related to Figure 2.**

**
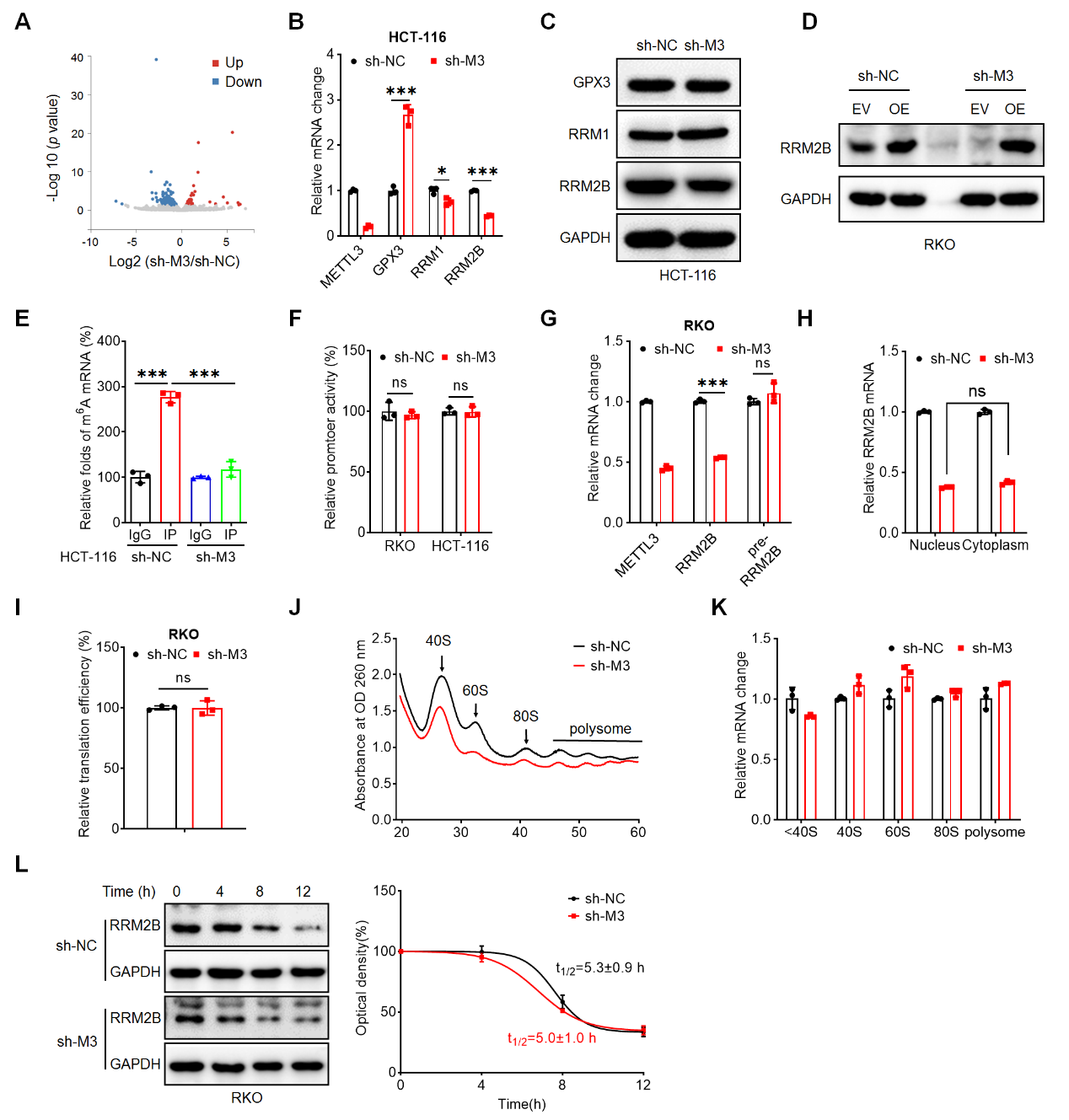
**

**Supplementary Figure 3 m^6^A regulated GSH synthesis through stabilization of *RRM2B* mRNA**

1. Volcano plots to determine different genes in sh-*METTL3* cells as compared with that in RKO cells. Each dot represents a gene;
2. The mRNA levels of *RRM2B*, *RRM1,* and *GPX3* in sh-control and sh-*METTL3* HCT-116 cells;
3. The protein expression of RRM2B, RRM1, and GPX3 in sh-control and sh-*METTL3* HCT-116 cells;
4. The protein expression of RRM2B in RKO cells transfected with vector control or RRM2B plasmid for 24 h;
5. m^6^A RIP-qPCR analysis of RRM2B in sh-control and sh-*METTL3* HCT-116 cells;
6. Cells were transfected with pGL3-Basic-*RRM2B*-luc reporter and pRL-TK plasmid for 24 h, the promoter activities were presented as the ratios of the reporter normalized to pRL-TK plasmid;
7. The levels of precursor *RRM2B* mRNA in sh-control and sh-*METTL3* RKO cells;
8. The relative levels of nuclear versus cytoplasmic *RRM2B* mRNA in sh-control and sh-*METTL3* RKO cells;
9. The translation efficiency of endogenous RRM2B in sh-control and sh-*METTL3* RKO cells;
10. Ribosome profiling of sh-control and sh-*METTL3* RKO cells;
11. The mRNA levels of *RRM2B* in non-ribosome portion (<40S), 40S, 60S, 80S, and polysome fractions in sh-control and sh-*METTL3* RKO cells.
12. Cells were treated with 10 μg/ml CHX for the indicated time periods, the protein expression of RRM2B was detected by western blot analysis (left) and quantitatively analyzed (right)

Data are presented as mean ± SD from three independent experiments. **p*<0.05, ***p*<0.01, ****p*<0.001, by Student’s *t* test between two groups and by one-way ANOVA followed by Bonferroni test for multiple comparison.

**Related to Figure 3.**

**
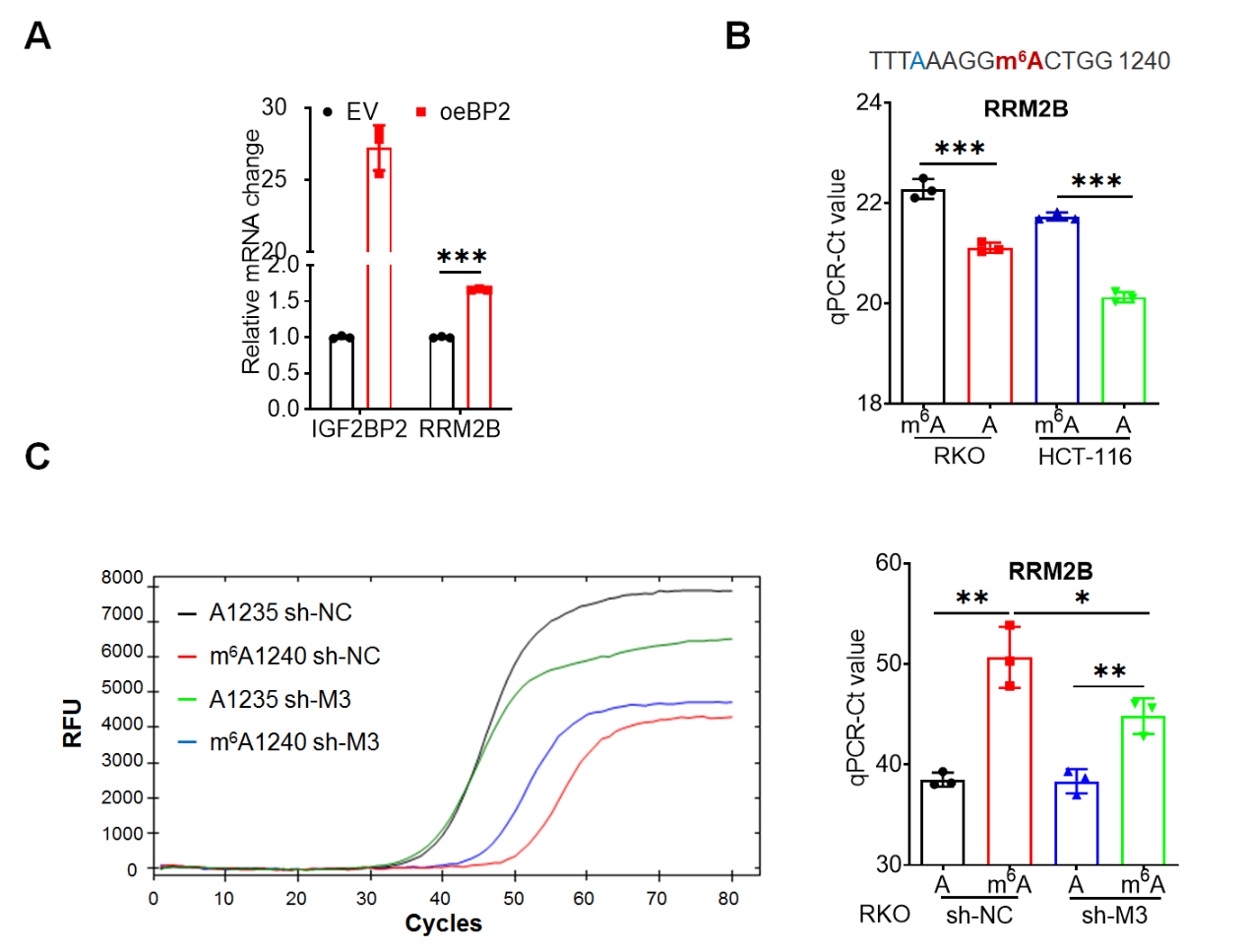
**

**Supplementary Figure 4 m^6^A stabilized *RRM2B* mRNA via methylation of A1240 at 3’UTR**

1. The mRNA levels of IGF2BP2 and RRM2B in sh-*METTL3* RKO cells transfected with vector control or IGF2BP2 plasmid for 24 h;
2. The threshold cycle (Ct) of qPCR showing SELECT results for detecting m^6^A site in the potential m^6^A site (A1240) and negative A site (A1235) of RRM2B in RKO and HC-T116 cells;
3. m^6^A-Rol-LAMP detection of methylation modified site (A1240) and negative site (A1235) of *RRM2B* mRNA in sh-control or sh-*METTL3* RKO cells.

Data are presented as mean ± SD from three independent experiments. **p*<0.05, ***p*<0.01, ****p*<0.001, by Student’s *t* test between two groups and by one-way ANOVA followed by Bonferroni test for multiple comparison.

**Related to Figure 4.**

**
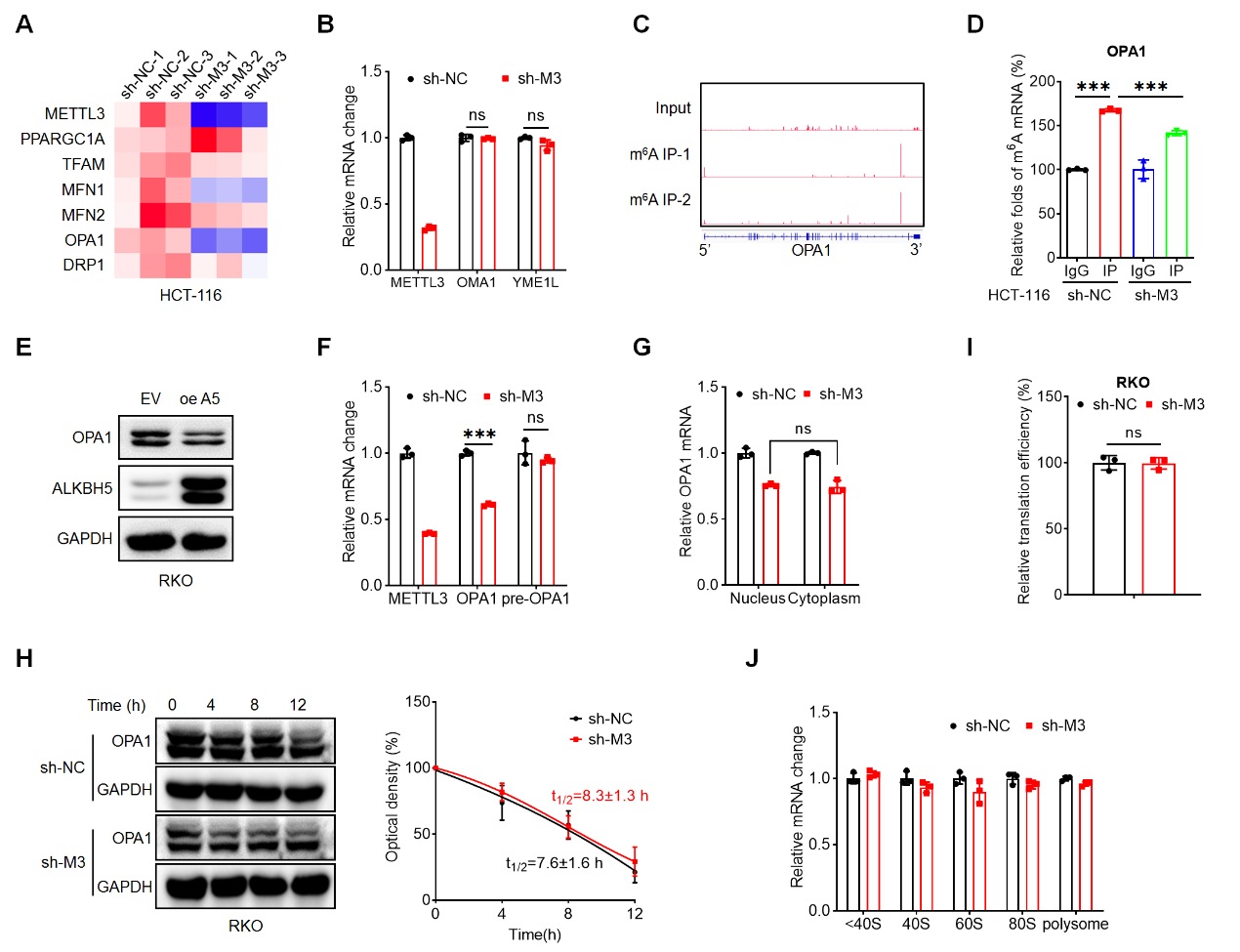
**

**Supplementary Figure 5 m^6^A-stablized *OPA1* mRNA regulated mitochondrial fusion**

1. Heatmap of mRNA expression of *PGC-1α* (peroxisome proliferator–activated receptor gamma coactivator 1-alpha, a transcriptional factor that regulates mitochondrial biogenesis); *TFAM*(transcription factor A, mitochondrial downstream of PGC-1α ); mitochondrial dynamic-related proteins, including *Mfn1* (mitofusin 1), *Mfn2* (mitofusin 2), *OPA1* (optic atrophy 1) and *Drp1* (dynamin-related protein 1), in sh-control and sh-*METTL3* HCT-116 cells;
2. The mRNA expression of OPA1 splicing related genes *OMA1* and *YME1L* in sh-control and sh-*METTL3* RKO cells;
3. m^6^A peaks of *OPA1* mRNA from m^6^A RIP-seq;
4. m^6^A RIP-qPCR analysis of *OPA1* in sh-control and sh-*METTL3* HCT-116 cells;
5. The protein expression of OPA1 in RKO cells transfected with vector control or ALKBH5 plasmid for 24 h;
6. The levels of precursor *OPA1* mRNA in sh-control and sh-*METTL3* RKO cells;
7. The relative levels of nuclear versus cytoplasmic *OPA1* mRNA in sh-control and sh-*METTL3* RKO cells;
8. Cells were treated with 10 μg/ml CHX for the indicated time periods, the protein expression of OPA1 was detected by western blot analysis (left) and quantitatively analyzed (right);
9. The translation efficiency of endogenous OPA1 in sh-control and sh-*METTL3* RKO cells;
10. The mRNA levels of OPA1 in non-ribosome portion (<40S), 40S, 60S, 80S, and polysome fractions in sh-control and sh-*METTL3* RKO cells.

Data are presented as mean ± SD from three independent experiments. ****p*<0.001, ns, no significant, by Student’s *t* test between two groups and by one-way ANOVA followed by Bonferroni test for multiple comparison.

**Related to Figure 5.**

**
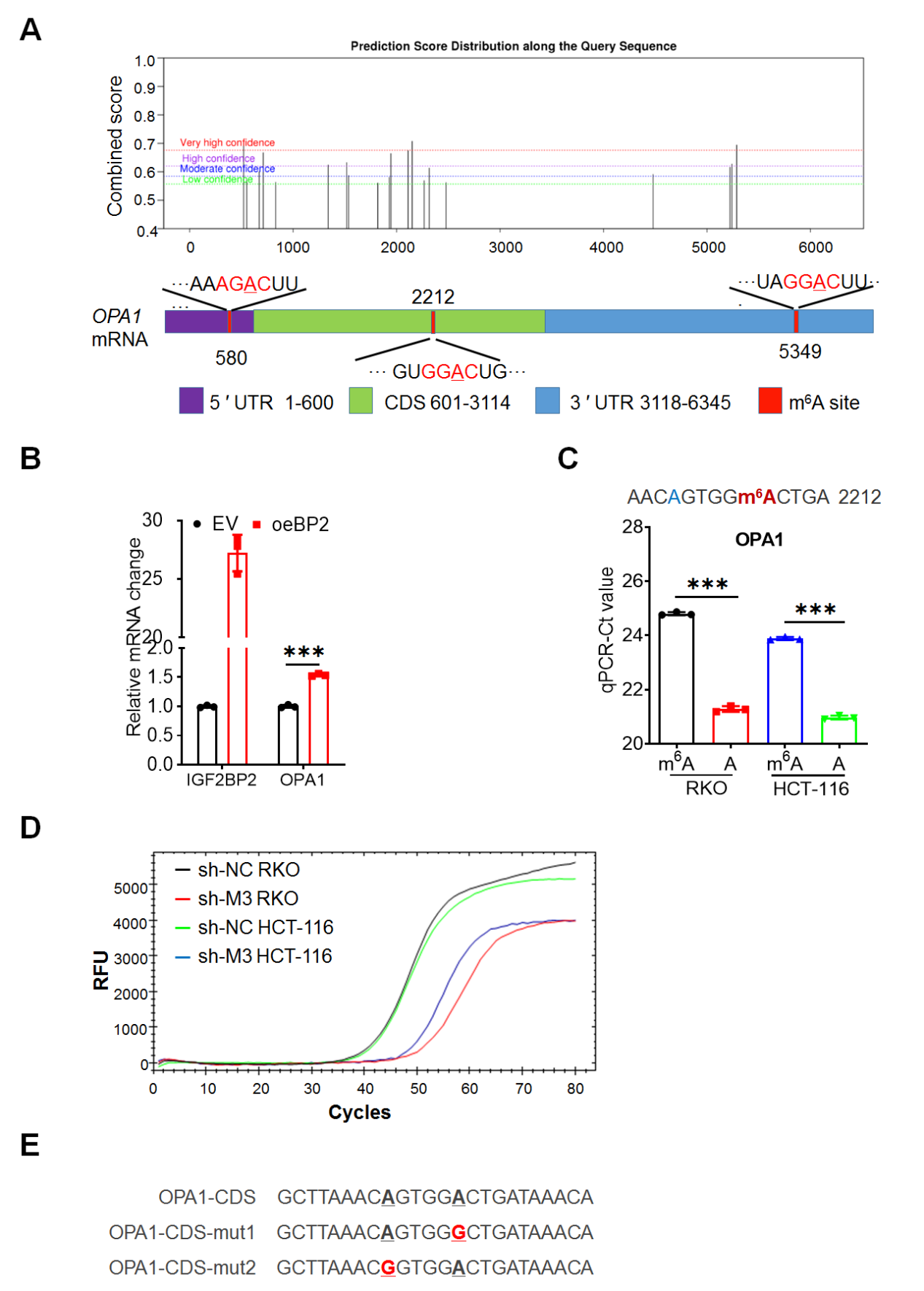
**

**Supplementary Figure 6 m^6^A methylation at A2212 stabilized *OPA1* mRNA via binding with IGF2BP2**

1. The predicted m^6^A peaks in *OPA1* mRNA from the m^6^A sites predictor SRAMP;
2. The mRNA levels of IGF2BP2 and OPA1 in sh-*METTL3* RKO cells transfected with vector control or IGF2BP2 plasmid for 24 h;
3. The threshold cycle (Ct) of qPCR showing SELECT results for detecting m^6^A site in the potential m^6^A site (A2212) and negative A site (A2207) of OPA1 in RKO and HC-T116 cells;
4. m^6^A-Rol-LAMP detection of methylation modified sites (A2212) of *OPA1* mRNA in sh-control or sh-*METTL3* RKO and HCT-116 cells;
5. Schematic representation of mutated (GGAC to GGGC) CDS of pmirGLO vector to investigate the roles of m^6^A in CDS in OPA1 expression.

Data are presented as mean ± SD from three independent experiments. ***p*<0.01, ****p*<0.001, by Student’s *t* test between two groups and by one-way ANOVA followed by Bonferroni test for multiple comparison.

**Related to Figure 6.**


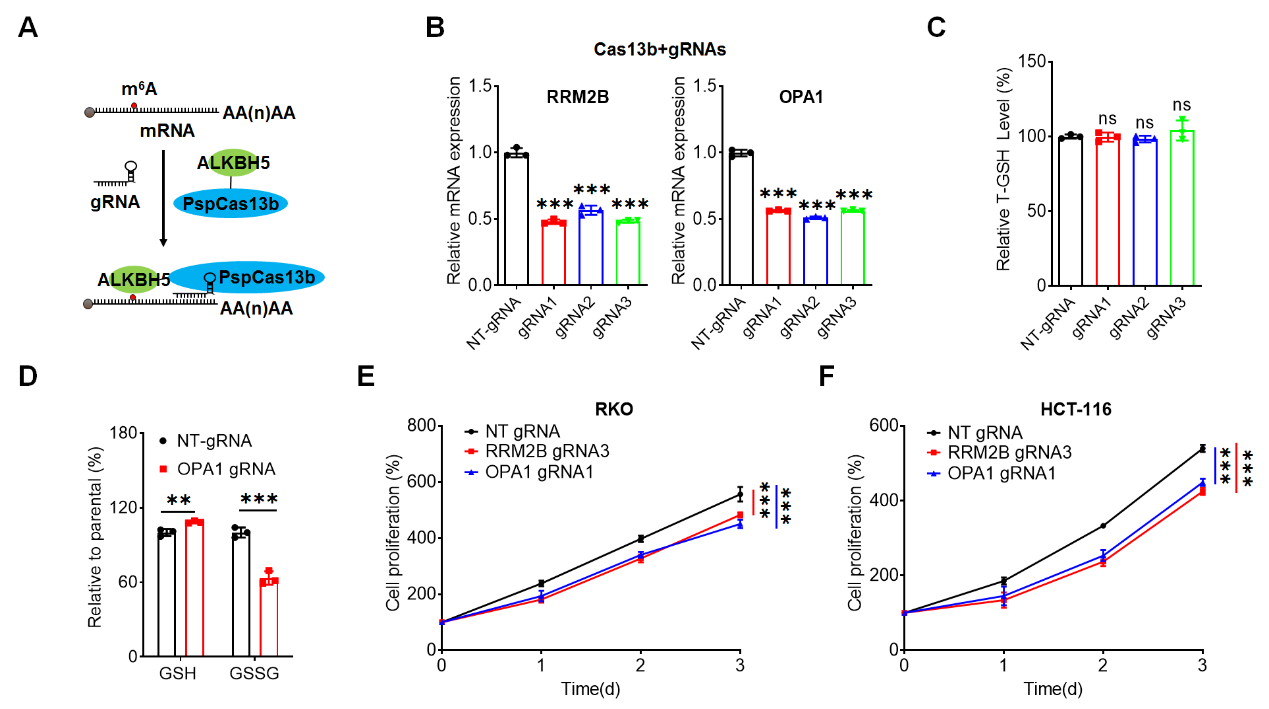


**Supplementary Figure 7 Generally or specifically targeting m^6^A suppressed mitochondria fusion.**

1. Overview of site-specific RNA targeting using dCas13b-guided fusion proteins with gRNA;
2. The mRNA levels of *RRM2B* and *OPA1* in RKO cells transfected with Cas13b combined with gRNA control or gRNA1/2/3, respectively, for 24 h;
3. The GSH levels in RKO cells transfected with gRNA control, gRNA1/2/3 for OPA1, and dCas13b-ALKBH5 for 24 h;
4. The GSH and GSSG levels in RKO cells transfected with gRNA negative control, gRNA3 for *OPA1*, and dCas13b-ALKBH5 for 24 h;

(E&F) The proliferation of RKO (E) and HCT-116 (F) cells transfected with dCas13b-ALKBH5 combined with NT-gRNA, RRM2B gRNA3 or OPA1 gRNA1, respectively, for the indicated time periods.

Data are presented as mean ± SD from three independent experiments. ****p*<0.001, ns, no significant, by Student’s *t* test between two groups and by one-way ANOVA followed by Bonferroni test for multiple comparison.

**Related to Figure 7.**

**
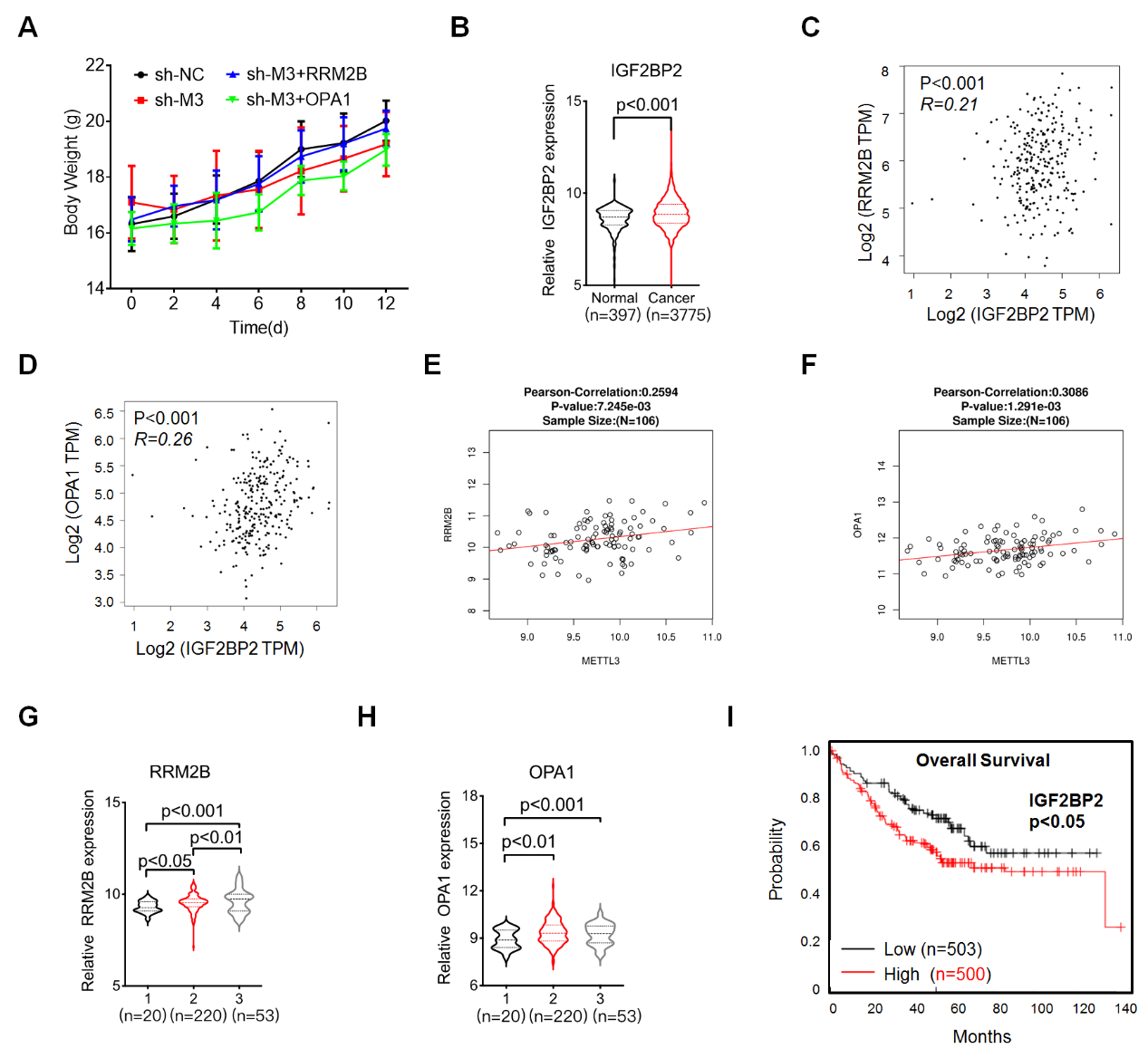
**

**Supplementary Figure 8 Oncogenic roles of m^6^A-regulated mitochondria dynamic in CRC development**

1. The body weight of mice with sh-control, sh-*METTL3*, and RRM2B or OPA1 stable over expression sh-*METTL3* RKO xenografts;
2. Expression of IGF2BP2 in CRC tumor tissues and adjacent normal mucosa tissues from GENT database;

(C&D)Correlation between IGF2BP2 and RRM2B (C) or OPA1 (D) in CRC patients from GEPIA database;

(E&F)Correlation between METTL3 and RRM2B (E) or OPA1 (F) in CRC patients from CPTAC database;

(G&H)Expression of RRM2B (G) or OPA1 (H) in grade 1/2/3 CRC tissues from GEPIA database;

(I) The Kaplan-Meier survival curves of OS based on IGF2BP2 in CRC cancer patients from TCGA database.

**Related to Figure 8.**

**Materials and methods**

- 1. **Cell line and cell culture**

Human colorectal cancer RKO and HCT-116 cell lines were commercially obtained from American Tissue Cell Culture (ATCC, USA) and maintained by our laboratory. Cells were cultured in high-glucose DMEM (GIBIO, USA) medium supplemented with 10% FBS (GIBIO, USA) and 100 U/ml penicillin/streptomycin (Beyotime, China) under an atmosphere of 5% CO2 at 37℃. The stable cell lines were cultured in medium containing puromycin or neomycin until 3 days before experiment.

- 1. **Plasmid, siRNA, shRNA and generation of stable cell lines**

The cDNA of RRM2B, OPA1 were cloned into the pcDNA3 vector (Invitrogen, USA), the CDS of ALKBH5 was cloned into pcDNA3.1 (Invitrogen, USA), and the CDS of METTL3 was cloned into the ppB vector to generate over expression plasmid, while the METTL3 mutant DA (D395A) plasmid was generated in our previous study ([1](#_ENREF_1)). The following siRNAs were synthesized (Ribobio, China) and used in the study: siRNA negative control (si-NC): 5’-UUC UCC GAA CGU GUC ACG U-3’; IGF2BP2 #1: 5’-CAT GCC GCA TGA TTC TTG A-3’; IGF2BP2 #2: 5’-GAA CGA ACT GCA GAA CTT A-3’; IGF2BP2 #3: 5’-AAC AGG GAC CAA GAT AAC A-3’. To generate stable cell lines with continuous suppression of METTL3, cells were transfected with lentivirus-shRNA for negative control, and METTL3, respectively, before selection with puromycin. For stable overexpression of pcDNA3/RRM2B and pcDNA3/OPA1, cells were transfected with plasmids by lipofectamine 3000 according to the manufacturers’ instructions (Invitrogen, USA) and selected with neomycin.

- 1. **Intracellular GSH, GSSG and ATP Measurements**

Intracellular GSH、GSSG levels were measured according to GSH and GSSG assay kit (Beyotime, China) according to the manufacturer’s instructions. Cellular ATP content was measured using a luciferin/luciferase-based kit (Beyotime, China) according to the manufacturer’s instructions.

- 1. **Flow cytometric analysis**

Mitochondrial membrane potential and mitochondrial mass were measured after cells were treated with Mito-Tracker Red CMXROS (Invitrogen, USA) or MitoTracker Deep Red (Invitrogen, USA) for 30 min at 37℃. Thereafter, cells underwent trypsinization, and then cell fluorescence was assessed by flow cytometry with a BD flow cytometry (BD Biosciences, USA). For apoptosis, an Annexin V Apoptosis Detection Kit (Santa-Cruz Biotechnology Inc., USA) was used according to the manufacturer’s instructions.

- 1. **Western blot analysis**

Cells were washed with PBS, lysed in radio-immunoprecipitation assay (RIPA) buffer containing 1 mM PMSF (Beyotime, China), and placed on ice for 30 min. Then, cells were centrifuged at 12,000×g for 20 min, and the protein concentration was determined using BCA Protein Assay Kit (Thermo Fisher, USA). Proteins were separated by 10% SDS-PAGE gel and electro-transferred to polyvinylidene difluoride membrane (Bio-Rad, USA). The membranes were blocked with 5% nonfat milk in 1×PBST for 30 min at room temperature, and incubated at 4℃ overnight with the following primary antibodies: anti-METTL3 (15073-1-AP, proteintech, China); anti-ALKBH5 (ab195377, Abcam, England); Anti-RRM2B (A8020, Abclonal Technology, China); anti-YTHDF2 (ab220163, Abcam, England); anti-YTHDF1 (ab99080, Abcam, England); Anti-RRM1 (A1152, Abclonal Technology, China); Anti-OPA1 (A9833, Abclonal Technology, China); Anti-IGF2BP1 (8482S, CST, USA); Anti-IGF2BP2 (14672S, CST, USA); Anti-IGF2BP3 (25864S, CST, USA); Anti-MFN1 (A9880, Abclonal Technology, China); Anti-MFN2 (A12771, Abclonal Technology, China); Anti-DRP1 (A2586, Abclonal Technology, China). Anti-GAPDH (5174Ss, CST, USA) was used as an internal loading control. After incubation with corresponding secondary antibodies (CST, USA), the membranes were incubated with ECL substrate (Thermo Fisher, USA).

- 1. **Metabolomics analysis**

Cells were washed with cold PBS and collected using a cell scraper. Then all cell samples were transferred into 2 ml EP tube, accurately added 1 ml of acetonitrile: methanol: H_2_O mixed solution (2:2:1, v/v/v), and vortex for 30 s. The tubes were placed into liquid nitrogen for 5 min and thawed at room temperature, then putted it into the tissue grinder for 2 min at 55 Hz, and repeated the operation twice. After centrifuged at 4℃ for 10 min at 12,000×g, samples were then transferred 850 µl of the supernatant from each sample into another 2 ml centrifuge tube. Samples were concentrated to dry in vacuum. Dissolved samples with 300 µL of 2-chlorobenzalanine solution (4 ppm) prepared with acetonitrile: 0.1% FA (1:9, v/v) (-20℃), and the supernatant was filtered through 0.22 µm membrane to obtain the prepared samples for LC-MS. Taken 20 µl from each sample to the quality control (QC) samples (These QC samples were used to monitor deviations of the analytical results from these pool mixtures and compare them to the errors caused by the analytical instrument itself), and then the samples were used for LC-MS detection.

For LC, Chromatographic separation was accomplished in an Thermo Vanquish system equipped with an ACQUITY UPLC® HSS T3 (150×2.1 mm, 1.8 µm, Waters) column maintained at 40℃. The temperature of the autosampler was 8℃. Gradient elution of analytes was carried out with 0.1% formic acid in water (A2) and 0.1% formic acid in acetonitrile (B2) or 5 mM ammonium formate in water (A3) and acetonitrile (B3) at a flow rate of 0.25 ml/min. Injection of 2 μl of each sample was done after equilibration. An increasing linear gradient of solvent B2/B3 (v/v) was used as follows: 0~1 min, 2% B2/B3; 1~9 min, 2%~50% B2/B3; 9~12 min, 50%~98% B2/B3; 12~13.5 min, 98% B2/B3; 13.5~14 min, 98%~2% B2/B3; 14~20 min, 2% B2-positive model (14~17 min, 2% B3-negative model).

For MS, the ESI-MSn experiments were executed on the Thermo Q Exactive mass spectrometer with the spray voltage of 3.5 kV and -2.5 kV in positive and negative modes, respectively. Sheath gas and auxiliary gas were set at 30 and 10 arbitrary units, respectively. The capillary temperature was 325℃. The analyzer scanned over a mass range of m/z 81-1 000 for full scan at a mass resolution of 70 000. Data dependent acquisition (DDA) MS/MS experiments were performed with HCD scan. The normalized collision energy was 30 eV. Dynamic exclusion was implemented to remove some unnecessary information in MS/MS spectra.

- 1. **RNA-extraction and real-time PCR**

Total RNA was isolated using TRIzol Reagent (Agbio, AG21102, China) and reversed by Evo M-MLV (Agbio, AG11706, China). qRT-PCR was assessed with SYBR Green II (Agbio, AG11701, China) using CFX Manager 3.1 (Bio-Rad, USA) as recommended by the manufacturer’s protocol. The primers used are listed at Table S5 in the Supplementary Data. GAPDH was used as a control for normalization. The relative gene expression levels were calculated using 2^−ΔΔCT^ method.

- 1. **Mitochondrial DNA assay**

Mitochondrial DNA (mtDNA) copy number was determined by a standard by real-time PCR using nuclear DNA (nDNA, GAPDH primer set) content as an internal control for normalization according to our previous study ([1](#_ENREF_1)). Extract genomic DNA (including mtDNA) from target cells were isolated using the Qiagen Genomic DNA kit according to the manufacturer’s instructions. Cells relative content of mtDNA (mtDNA primer set) was checked by qRT-PCR and normalized to that of nuclear DNA (GAPDH primer set). The primers were as follow: mtDNA, forward 5’-ACG CCA TAA AAC TCT TCA CCA AAG-3’, reverse 5’-GGG TTC ATA GTA GAA GAG CGA TGG-3’; GAPDH, forward 5’-ACA ACT TTG GTA TCG TGG AAG G-3’, reverse 5’-GCC ATC ACG CCA CAG TTT C-3’.

- 1. **m^6^A-RIP qPCR**

We performed m^6^A qPCR using Magna MeRIP™ m^6^A Kit (Millipore, MA) in accordance with manufacturer’s protocol. Briefly, 200 μg total RNA was isolated and randomly fragmented with chemical reagents treatment followed by the immunoprecipitation with 5 μg m^6^A antibody or mouse IgG which was linked to Magna ChIP Protein A/G Magnetic Beads. After extensive washing with IP Buffer, the beads were treated with proteinase K for 30 min at 55℃ with occasional shaking. RNA was purified from the supernatant using TRIzol Reagent following the manufacturer’s instructions. Interested mRNA levels in the elutes were measured by RT-qPCR.

- 1. **RIP-RT-PCR**

Two 10-cm plates of cells were washed twice with cold PBS before collected. 400 μl IP lysis buffer (150 mM KCl, 25 mM Tris (pH 7.4), 5 mM EDTA, 0.5 mM DTT, 0.5% NP40, 1×protease inhibitor, 1 U/μl RNase inhibitor) was added and resuspended it on ice. The lysate was centrifuged at 12,000×g for 10 min. Then Magnetic beads pre-coated with 4 μl targeted antibodies or mouse IgG (NEB, USA) were incubated with sufficient cell lysates at 4℃ overnight. The beads containing immunoprecipitated RNA-protein complex were treated with proteinase K to remove proteins. Then interested RNAs were purified by TRIzol methods and detected by RT-qPCR with the normalization to input.

- 1. **Sub-cellular fraction**

Fractionation of nuclear and cytoplasmic samples was conducted using Nuclear and Cytoplasmic Extraction Kit (Beyotime, China) according to the manufacturer’s guidelines.

- 1. **Polysome profiling**

The fraction of ribosome was separated by centrifugation in a sucrose gradient according to our previous study ([1](#_ENREF_1)).

- 1. **Protein stability**

To measure protein stability, cells were seeded in 6-well plates and treated with cycloheximide (CHX, Catalog #14126, Cayman, USA) at final concentration 20 μg/ml during indicated times. Cells were collected and lysed in lysis buffer. The expression of proteins was measured through western blot analysis.

- 1. **mRNA stability**

To measure RNA stability in tumor cells, actinomycin D (Act-D, Catalog #A9415, Sigma, USA) at 10 μg/ml was added to cells in 6-well plates. After incubation at the indicated times, cells were collected, and RNA was isolated for real-time PCR. Half-life (t_1/2_) of mRNA were calculated using ln2/slope and 18S was used for normalization.

- 1. **SELECT**

SELECT qPCR was conducted by following Xiao’s protocol ([2](#_ENREF_2)) and our previous study([3](#_ENREF_3)). Briefly, 1500 ng of total RNA was mixed with 40 nM up and down primers and 5 μM dNTP in 17 μl 1×CutSmart buffer (NEB, China). The mixture was incubated with the follow program: 90℃ for 1 min, 80℃ for 1 min, 70℃ for 1 min, 60℃ for 1 min, 50℃ for 1 min and 40℃ for 6 min. The sample was further mixed with 0.5 U SplintR ligase, 10 nM ATP and 3 μl of 0.01 U Bst 2.0 DNA polymerase and incubated at 40℃ for 20 min and denatured at 80℃ for 20 min. Afterwards, 20 μl qPCR reaction containing 2 μl of final reaction mixture, 2×SYBR Green Master Mix (Takara, Japan), and 200 nM SELECT primers was performed. The qPCR program was 95℃, 5 min; (95℃, 10 s; 60℃, 35 s)×40 cycles; 95℃, 15 s; 60℃, 1 min; 95℃, 15 s; 4℃ hold. Results were calculated by normalized the Ct values of samples to their corresponding Ct values of control. All assays were performed with three independent experiments. Primers for SELECT qPCR were listed in Table S5.

- 1. **m^6^A-Rol-LAMP**

Total RNA was mixed with 25 pmol padlock probes and hybridized in 8 μl 1×CutSmart buffer (NEB, China) in 95℃ for 1 min, 80℃ for 1 min, 70℃ for 1 min, 60℃ for 1 min, 50℃ for 1 min, 40℃ for 1 min, 30℃ for 10 min. Then, 2 μl mixture containing 0.05 U Bst 2.0 DNA polymerase, 0.5 U SplintR, 10 nM ATP and 5 μM dNTP were added to hybridization products and incubated at 37℃ for 30 min, 65℃ for 10 min and kept at 4℃. 2 μl ligation products was used for Rol-LAMP amplification detected by RT-qPCR. Then, 20 μl m^6^A-Rol-LAMP reaction was set up as followed: 2 μl ligation products, 0.8 μM FIP, 0.8 μM BIP, 100 nM SLP, 1.4 mM dNTP, 6 mM MgSO4, 1×Isothermal Amplification Buffer (20 mM Tris-HCl, 10 mM (NH4)_2_SO_4_, 50 mM KCl, 2 mM MgSO_4_, 0.1% Tween® 20, pH 8.8), and 0.16 U/μl Bst 2.0 DNA polymerase. Reactions were performed at 65℃. Reaction time was determined by RNA samples. 9 μl of m^6^A-Rol-LAMP product was mixed with 0.5 μl SYBR Green I and then recorded.

- 1. **Design of the guide RNAs for dm^6^ACRISPR**

The guide RNAs of RRM2B and OPA1 for dm^6^ACRISPR was designed according to our previous study ([3](#_ENREF_3)).

- 1. **Xenograft models**

BALB/c nude mice (4 weeks old) were purchased from Sun Yat-sen University Animal Center and raised under pathogen-free conditions. The experimental handling and care procedures for the mice were approved by the Animal Experimentation Ethics Committee of Sun Yat-sen University (No. 2022001784). For subcutaneous transplanted model, sh-control, sh-*METTL3* and sh-*METTL3*+RRM2B, sh-*METTL3*+OPA1 stable over expression RKO cells (5 × 10^6^ per mouse, n = 5 for each group) were diluted in 100 μl of PBS and subcutaneously injected into immunodeficient mice to investigate tumor growth. Tumor growth was monitored every two days. When tumor volume grew appropriately, mice were killed, and tumors were removed and weighed for use in histology and further studies. The tumor volume was calculated using the formula V = 1/2× larger diameter × (smaller diameter) ^2^.

- 1. **Database (DB) search**

We used Kaplan-Meier database (http://kmplot.com/analysis/) to test overall survival (OS) of METTL3/RRM2B axis. Difference between survival curves was determined by log-rank test that its *p* value. Correlations between METTL3 protein expression in CRC tissues and other proteins such as RRM2B were extracted from CPTAC database. The mRNA expression of METTL3 and RRM2B in CRC and normal tissues were analyzed in the GENT database (http://medicalgenome.kribb.re.kr/GENT/), followed by analyzation and visualization with GraphPad Prism. The data was analyzed with Pearson Chi-square (χ2) test then.

**Supplementary References**

1. Li, ZH, Peng, YX, Li, JX*, et al.* N-6-methyladenosine regulates glycolysis of cancer cells through PDK4. *Nat Commun*. 2020; **11**(1): 2578

2. Xiao, Y, Wang, Y, Tang, Q*, et al.* An Elongation- and Ligation-Based qPCR Amplification Method for the Radiolabeling-Free Detection of Locus-Specific N(6) -Methyladenosine Modification. *Angew Chem Int Ed Engl*. 2018; **57**(49): 15995-6000.

3. Li, J, Chen, Z, Chen, F*, et al.* Targeted mRNA demethylation using an engineered dCas13b-ALKBH5 fusion protein. *Nucleic Acids Res*. 2020; **48**(10): 5684-94.
